# The quaternary state of polymerized human hemoglobin regulates oxygenation of breast cancer solid tumors: A theoretical and experimental study

**DOI:** 10.1371/journal.pone.0191275

**Published:** 2018-02-07

**Authors:** Donald A. Belcher, Julia A. Ju, Jin Hyen Baek, Ayla Yalamanoglu, Paul W. Buehler, Daniele M. Gilkes, Andre F. Palmer

**Affiliations:** 1 William G. Lowrie Department of Chemical and Biomolecular Engineering, The Ohio State University, Columbus, OH, United States of America; 2 Department of Chemical and Biomolecular Engineering, The Johns Hopkins University, Baltimore, MD, United States of America; 3 Division of Blood Components and Devices, Laboratory of Biochemistry and Vascular Biology, FDA/CBER, Silver Spring, MD, United States of America; 4 Department of Oncology, The Johns Hopkins University School of Medicine, Baltimore, MD, United States of America; University of Tennessee Health Science Center, UNITED STATES

## Abstract

A major constraint in the treatment of cancer is inadequate oxygenation of the tumor mass, which can reduce chemotherapeutic efficacy. We hypothesize that polymerized human hemoglobin (PolyhHb) can be transfused into the systemic circulation to increase solid tumor oxygenation, and improve chemotherapeutic outcomes. By locking PolyhHb in the relaxed (R) quaternary state, oxygen (O_2_) offloading at low O_2_ tensions (<20 mm Hg) may be increased, while O_2_ offloading at high O_2_ tensions (>20 mm Hg) is facilitated with tense (T) state PolyhHb. Therefore, R-state PolyhHb may deliver significantly more O_2_ to hypoxic tissues. Biophysical parameters of T and R-state PolyhHb were used to populate a modified Krogh tissue cylinder model to assess O_2_ transport in a tumor. In general, we found that increasing the volume of transfused PolyhHb decreased the apparent viscosity of blood in the arteriole. In addition, we found that PolyhHb transfusion decreased the wall shear stress at large arteriole diameters (>20 μm), but increased wall shear stress for small arteriole diameters (<10 μm). Therefore, transfusion of PolyhHb may lead to elevated O_2_ delivery at low pO_2_. In addition, transfusion of R-state PolyhHb may be more effective than T-state PolyhHb for O_2_ delivery at similar transfusion volumes. Reduction in the apparent viscosity resulting from PolyhHb transfusion may result in significant changes in flow distributions throughout the tumor microcirculatory network. The difference in wall shear stress implies that PolyhHb may have a more significant effect in capillary beds through mechano-transduction. Periodic top-load transfusions of PolyhHb into mice bearing breast tumors confirmed the oxygenation potential of both PolyhHbs via reduced hypoxic volume, vascular density, tumor growth, and increased expression of hypoxia inducible genes. Tissue section analysis demonstrated primary PolyhHb clearance occurred in the liver and spleen indicating a minimal risk for renal damage.

## Introduction

A major constraint that continues to limit the effectiveness of traditional chemotherapy and other cancer therapies is inadequate oxygenation of the tumor mass. Hypoxia and the subsequent production of hypoxia-inducible factors (HIFs) reduces the effectiveness of chemotherapeutic agents by promoting drug resistance [1–4]. In normal tissue, hypoxia induces apoptosis or necrosis [5]. However, cancer cells can adapt to hypoxic conditions by increasing the production of HIF-1α and HIF-2α [6]. These proteins activate genes that promote cell survival, metabolic reprogramming, stem cell maintenance, metastasis, immortalization, and the epithelial-mesenchymal transition [7,8]. In previous work, inhibition of HIFs via RNA interference limited tumor growth and reduced spontaneous metastasis in mouse models of breast cancer [9–12]. Due in part to HIF expression, many cancers are unresponsive to traditional cytotoxic chemotherapies under hypoxic conditions.

**Fig 1:**
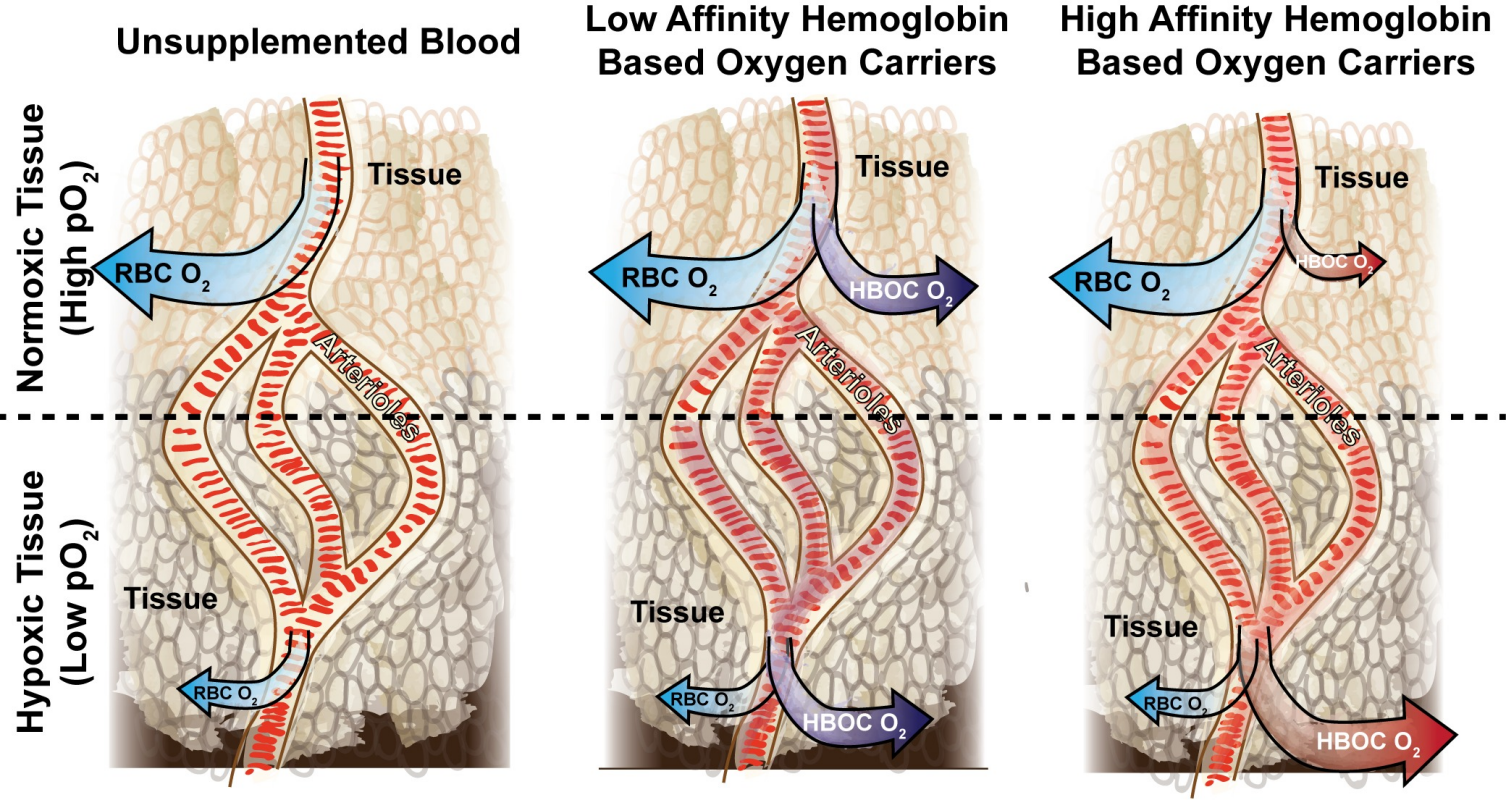
Illustration of predicted performance of transfused high and low O_2_ affinity HBOCs compared to a control of unsupplemented blood. The magnitude of O_2_ delivery to the tissue from each species is designated by the size of the arrow. Note that O_2_ delivery should be elevated at all conditions with low O_2_ affinity HBOCs. In contrast, O_2_ delivery is elevated under hypoxic conditions for high O_2_ affinity HBOCS.

Systemic administration of an O_2_ carrier can increase O_2_ delivery under hypoxic conditions. Hemoglobin (Hb)-based O_2_ carriers (HBOCs) can deliver O_2_ to tumor tissues following intravenous administration. HBOCs are currently in development as oxygen carriers, primarily in patients where blood is contraindicated [13]. Previously studied HBOCs include polymerized Hb, cross-linked Hb, polyethylene glycol (PEG) conjugated Hb, and liposome encapsulated Hb. Each of these materials demonstrated the ability to increase O_2_ delivery to tumor tissue, and improve the effectiveness of anti-cancer therapeutics [14–20]. In addition, increased oxygenation stemming from the transfusion of HBOCs such as polymerized human Hb (PolyhHb) may result in more normalized vessel growth by reducing VEGF induction and neo vessel formation [21]. Furthermore, increased tumor oxygenation may lead to decreased tumor growth due to the increased survival rate of normal cells. Most notably, the majority of HBOCs used in these tumor studies exhibited low O_2_ affinities. High O_2_ affinity HBOCs demonstrated limited potential to reduce the tumor hypoxic volume in a 1996 study [22]. In addition, moderate tumor oxygenation potential was observed with high affinity HBOCs in a study conducted in 1998 [23]. A 2005 study demonstrated that low O_2_ affinity PolyhHb oxygenated a rhabdomyosarcoma to a lesser extent than carbogen-based therapies [24]. In contrast, a 2008 computational simulation indicated that HBOCs with reduced O_2_ affinities may selectively oxygenate hypoxic tissue [25]. The predicted performance of low and high O_2_ affinity PolyhHbs are shown in Fig 1. Because of mixed performance observed in some animal models, there are ongoing concerns regarding the effectiveness of HBOCs for solid tumor oxygenation.

Initial pre-clinical and clinical trials of HBOCs suggested the potential for adverse events consistent with hypertension and oxidative tissue damage [26–32]. These toxic effects are associated with nitric oxide (NO) scavenging by the HBOC, heme release, heme oxidation, and HBOC extravasation into the tissue space [33–35]. We have recently explored the biophysical properties of both high and low O_2_ affinity HBOCs via the production of polymerized Hbs locked in either the relaxed (R) or tense (T) quaternary state [36–38]. We have used information from prior studies and a recent down selection study to better engineer polymerized Hbs with decreased *in vivo* oxidation and increased circulatory half-life [39,40].

To assess the O_2_ delivery potential of PolyhHb, we computationally modeled a tumor arteriole with a modified Krogh tissue cylinder (KTC) model. In this model, we evaluated the effect of Starling flow across the blood vessel wall coupled with PolyhHb mass transport populated with PolyhHb O_2_ offloading kinetics, diffusivity, and rheological properties experimentally obtained from biophysical analysis of the synthesized PolyhHb molecules. Because we have engineered the size of the PolyhHb to reduce Hb extravasation mediated side effects, the therapeutic benefits of our PolyhHbs are likely coupled to perfusion in the tumor microcirculatory system. To assess how the PolyhHbs may respond in a variety of environments, we also explored how changes to vascular architecture, fluid flow, and tissue parameters affect O_2_ mass transport and flux into the tumor tissue. The modified KTC model we use in this study is not a perfect predictor of PolyhHb performance in the tumor microcirculatory system. However, the application of this model assists our understanding of a top-load transfusion of PolyhHb on oxygenation and hemodynamics within tumor arterioles. Finally, to evaluate the effectiveness of PolyhHb treatment in an *in vivo* environment, we analyzed tumor growth, intratumoral hypoxia, the expression of hypoxia-inducible genes as well as angiogenic capacity using a mouse model of triple negative breast cancer. Additionally, we assessed the kidney, liver and spleen for iron exposure and potential adverse tissue changes.

## Materials and methods

### PolyhHb enhanced plasma viscosity

PolyhHbs were synthesized from human Hb and glutaraldehyde using our previously reported procedure [40]. Expired human plasma for viscosity analysis was generously donated by Transfusion Services, Wexner Medical Center, The Ohio State University, Columbus, Ohio. The rheology of PolyhHb plasma mixtures was measured using a DV3T-CP cone and plate Rheometer (Brookfield AMETEK, Middleboro, MA) with cone spindle CPA-40Z. PolyhHb was mixed with human plasma at 0.1, 0.2, and 0.3 PolyhHb to plasma volume fractions. For all measurements, 0.5 mL of solution was placed in the rheometer. While monitoring the shear stress, the shear rate was increased from 200 to 850 s^-1^ for human plasma, 1 to 400 s^-1^ for PolyhHb mixtures, and 1 to 100 s^-1^ for pure PolyhHb solutions. For all rheological measurements, the sample cup temperature was maintained at 37°C. The resulting shear stress (τ) and shear rate (γ) were then fit to the power law equation (Eq 1) in Igor Pro v. 6.36 (Wavemetrics, Lake Oswego, OR) to yield the flow consistency index (*K*) and the flow behavior index (*n*_*f*_).

$$\tau =K{\left(\gamma \right)}^{n_{f}}$$ (1)

### Mathematical model: Assumptions and limitations

**Fig 2:**
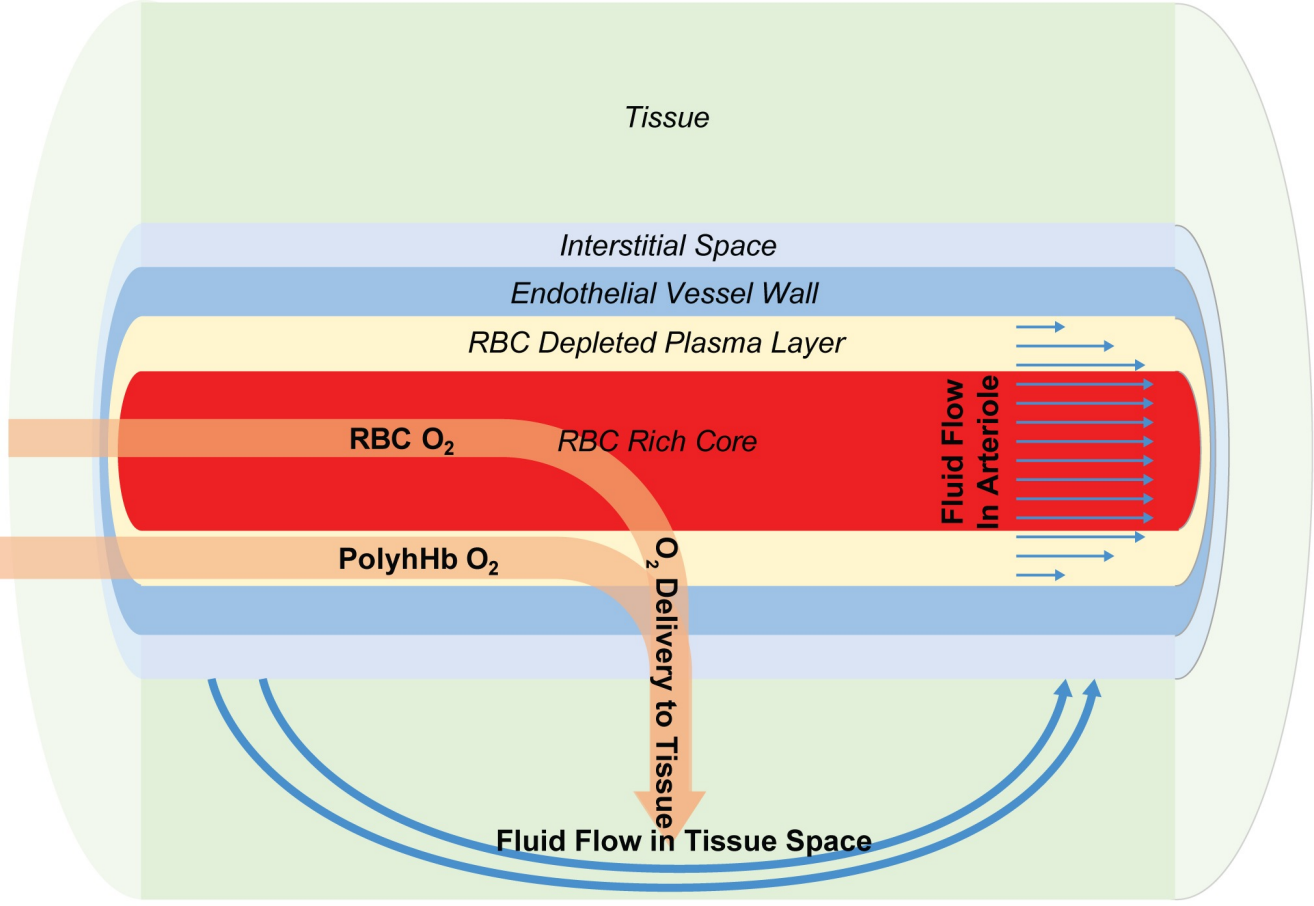
Schematic of the tumor arteriole KTC model.

To assess the effect of PolyhHb on hemodynamics and tissue oxygenation, we computationally modeled a tumor arteriole with a modified KTC model. The modified KTC model consists of four layers. The cylindrical core represents the RBC rich core of an arteriole. Moving away from the core, the first annulus models the RBC depleted plasma layer (i.e. cell-free layer), the second annulus models the endothelial vessel wall, the third annulus models the interstitial space, and the final annulus models the tissue space. A schematic of the model is shown in Fig 2. For the modified KTC model, the following assumptions were made: (1) Parallel arterioles were considered to be evenly distributed with negligible interactions between neighboring arterioles; (2) The system is axisymmetric; (3) The total PolyhHb concentration in the plasma is uniform at the inlet of the lumen; (4) Cells are uniformly distributed in the endothelium and in the tissue space. (5) All fluid enters and exits the system through the lumen; and (6) The RBC core behaves consistent with the Fahraeus-Lindqvist effect [41]. Like all KTC models, this model is limited by the simplified geometry [42] that alone does not represent the complete tumor microenvironment. The potential for heterogeneity is addressed with our sensitivity analysis. This model does not consider flow that can occur between neighboring blood vessels, assumes steady-state conditions and cannot fully account for biophysical descriptions, such as cellular response.

### Momentum transport mathematical model

Unlike previously developed HBOC transfusion KTC models, our model includes fluid flow using experimentally determined parameters and constitutive laws. Momentum transport partial differential equations were used to evaluate the velocity profile in all regions of the model. Here, the flow in the RBC rich core and the cell free layer is computed with the Navier-Stokes equation shown in Eq 2. Flow through the endothelial vessel wall, interstitial space, and tissue space is calculated using Brinkman’s equation shown in Eq 3.

$$\rho (\bar{v}∙\bar{\nabla })\bar{v}=[-\bar{\nabla }P+\mu \nabla^{2}\bar{v})$$ (2)

$$\bar{v}=\frac{\kappa }{\mu }\bar{\nabla }(-P)+\kappa \nabla^{2}\bar{v}$$ (3)

Where ρ, $\bar{v}$, μ, κ, and P represent the fluid density, fluid velocity, fluid viscosity, membrane intrinsic permeability, and pressure, respectively. The thickness of the cell free layer has been approximated as a function of the hematocrit and radius of the capillary from data generated by Fedesov *et al*. [43]. This relationship is shown in Eq 4.

$$r_{c}=2.06e^{-4.62H_{t}}\ln\;\left(r_{t}+7.65\right)+1.05$$ (4)

Where H_t_ and r_t_ correspond to the inlet hematocrit and the radius of the arteriole. The effective viscosity in the RBC rich core is computed with the Quemada constitutive law shown in Eq 5 [44].

$$\mu_{eff}=\frac{\tau }{\gamma }=\mu_{p}{\left(1-\frac{kH_{t}}{2}\right)}^{-2}$$ (5)

Where γ, τ, μ_p_, and H_c_ represent the shear rate, shear stress, plasma viscosity, and core hematocrit, respectively. The coefficient *k* is described by Eq 6.

$$k=\frac{k_{0}+k_{\infty }\sqrt{\gamma /\gamma_{c}}}{1+\sqrt{\gamma /\gamma_{c}}}$$ (6)

Where k_0_, k_∞_, and γ_c_ represent the maximum volume fraction for no shear rate, the maximum volume fraction for infinite shear rate, and the characteristic shear rate for RBC rouleau formation/ degradation, respectively. These values are estimated from the core hematocrit as described in Sriram *et al*. [45]. The core hematocrit in this model is calculated as a volumetric reduction of the total hematocrit as shown in Eq 7.

$$H_{c}=H_{t}\frac{R_{t}^{3}}{R_{c}^{3}}$$ (7)

In the simulations presented herein we apply a top load model of transfusion. For this model, we simulate a transfusion volume of PolyhHb equal to a set percentage of the total blood volume (TL%). The volume addition for transfusion can then be applied to calculate the effective total hematocrit and total PolyhHb concentration in the plasma (C_PolyhHb,total_) following top-load transfusion. These values are determined by applying the following equations.

$$H_{t}=\frac{H_{t,i}}{100+TL\%}$$ (8)

$$C_{{PolyhHb}_{total}}=C_{polyhHb_{i}}∙\frac{TL\%}{100+TL\%-H_{t,i}}$$ (9)

Where H_t,i_ is the initial hematocrit before top load transfusion (expressed as a percentage) and C_PolyhHb,i_ is the concentration of PolyhHb in the stock solution.

### Finite element model

To analyze this system of partial differential equations, a finite element model was implemented in COMSOL Multiphysics v 5.2 (COMSOL, Inc, Burlington MA). For this model, momentum transport was calculated using the free and porous media flow physics module. Free flow was permitted in the core and porous media flow was assigned to the capillary wall and tissue space. Mass transport of hHb in RBCs and PolyhHb was determined with the transport of dilute species physics module. Mass transport of O_2_ was calculated with dilute species transport properties in the blood vessel lumen and porous media transport elsewhere using the transport of dilute species in porous media physics module. The momentum transport equations were solved independently. The resulting velocity profiles were then incorporated into the mass transport equations, which were then solved. For all physics, axial symmetry is assumed around the axis of rotation (r = 0). Flow in the lumen was solved with a laminar flow module. The velocity profile at the inlet and outlet were set to a laminar flow distribution with a 50 μm entrance length. At the boundary between the lumen and the endothelial cell wall, the outlet pressure from the Navier-Stokes flow was continuous with the pressure calculated from the flow in porous media module. A no-slip Dirichlet boundary condition is placed along the exterior of the tissue space. A set concentration of O_2_ is specified at the inlet of the lumen. The concentration of both oxygenated Hb and PolyhHb are specified at the inlet given equilibrium values from the Hill equation. For O_2_, continuity is observed throughout the model. A no-flux boundary condition is specified at the exterior of the tissue space. For PolyhHb, a no-flux boundary condition is specified at the boundary between the lumen and the endothelial cell wall. At the lumen exit, both PolyhHb and O_2_ are given standard outflow conditions coupled to the laminar flow in the lumen. Continuity is assumed for all other boundaries. A 50 μm arteriole length was selected to help approximate flow behavior in short blood vessel segments. Because the microcirculatory architecture of tumors displays elevated fractal dimensions [46], modelling very long straight sections may inaccurately represent tumor O_2_ delivery. Additional information on the model parameters and mesh geometry for this model can be found in the S1 Text.

### Apparent viscosity

The effect of PolyhHb on the apparent viscosity of the blood/PolyhHb mixture in arteriole segments was assessed with the Hagen-Poiseuille equation shown in Eq 10.

$$\mu =\frac{\pi {r_{t}}^{4}\Delta P}{8LQ}$$ (10)

Where ΔP is calculated by taking the average fluid pressure at the inlet of the arteriole subtracted by the average fluid pressure at the outlet of the arteriole.

### O_2_ consumption rate

The amount of O_2_ lost from the arteriole to the surrounding tumor tissue was calculated by performing a mass balance on O_2_ in the arteriole space normalized by the tissue volume as described in Eq 11.

$$OCR=(C_{O_{2},total,in}-C_{O_{2},total,out})∙\frac{Q}{V_{tis}}$$ (11)

Where C_O2,total,in_ and C_O2,total,out_ represent the total concentration of O_2_ dissolved in plasma, bound to Hb inside RBCs and bound to PolyhHb at the inlet and outlet of the arteriole, respectively. To obtain these values, we evaluated a surface boundary integral on the dissolved O_2_, oxygenated Hb in RBCs, and oxygenated PolyhHb in COMSOL Multiphysics at the inlet and outlet of the arteriole.

### Overall O_2_ transport rate

The overall O_2_ transfer rate is an indicator of how each PolyhHb behaves when releasing O_2_. This parameter is calculated by taking the arteriole normalized O_2_ flux through the blood vessel wall as shown in Eq 12.

$$k_{0}=\frac{{\bar{J}}_{w}}{C_{O_{2},c}^{v}-C_{O_{2},w}^{s}}$$ (12)

Where ${\bar{J}}_{w}$ is the average O_2_ flux through the arteriole wall surface. $C_{O_{2},c}^{v}$ is the average concentration of dissolved O_2_ in the plasma obtained from a volume integral on the dissolved O_2_ in the arteriole. $C_{O_{2},w}^{s}$ is the surface averaged concentration of dissolved O_2_ in plasma at the arteriole wall surface.

### Computational estimate of hypoxic volume

The primary goal of PolyhHb transfusion into the recipient is to increase tumor oxygenation and thus decrease tumor hypoxia. To assess the effectiveness of both the 35:1 T-state PolyhHb and the 30:1 R-state PolyhHb in decreasing the tumor hypoxic volume, we calculated the minimum pO_2,in_ to achieve normoxic behavior throughout the mass of the tumor at varying tissue thicknesses. For this study, the pO_2_ hypoxic threshold was set at 5 mm Hg. Therefore, regions where the pO_2_ < 5 mm Hg, where considered to be hypoxic. To approximate the effect of PolyhHb clearance over time, we also varied the concentration of PolyhHb in the plasma, while maintaining the reduced hematocrit after top load transfusion. The thickness of the tissue layer was also varied to approximate the effect of varying blood vessel density within the tissue space.

### Computational model sensitivity to varied parameters

While the modified KTC model outlined here may be good descriptor of PolyhHb enhanced O_2_ transport in a single arteriole, the scope of this analysis is limited. In the tumor microenvironment, the microcirculatory network is heterogeneous with varying levels of fluid flow throughout the tumor tissue. Because of this, assumptions such as equal distribution of capillaries and uniform distribution of cells in the tissue space required in the KTC model do not fully represent the tumor microenvironment. To observe how varying model parameters may influence the OCR and k_0_, we scanned the parameters shown in Table 1 in a full factorial design. Due to the size of this data set, a response screening analysis was performed in JMP v. 12.2.0 (SAS Institute, Cary, NC). The log worth of the false detection rate (FDR) was selected to compare the magnitude of each effect at varying pO_2,in_. We also assessed if increasing the parameter led to an overall positive, negative, or mixed effect on the OCR and k_0_.

**Table 1:** Varied parameters for model sensitivity analysis.

Parameter	Min	Base	Max	Unit
%TL	10	20	30	%
V_M_	20	50	80	μM/s
K_M_	1	5	9	mm Hg
V_avg_	0.01	0.1	1	cm/s
R_c_	7	10	15	Mm
H_t_	0.3	0.45	0.6	
C_HBOC_	80	100	120	mg/mL

Each parameter in the analysis was varied to include the minimum and maximum values from the baseline value with accompanying units.

### Animal studies

Female 5- to 7-week-old NOD-SCID (Charles River Laboratories, Wilmington, MA) mice were used according to protocols approved by the Johns Hopkins University Animal Care and Use Committee. Mice were anesthetized with ketamine and xylazine injection, and 2 × 10^6^ MDA-MB-231 (human metastatic mammary carcinoma cells) resuspended in a 50:50 PBS:Matrigel solution were injected into the mammary fat pad. MDA-MB-231 cells were obtained from the American Type Culture Collection and cultured in DMEM media supplemented with 10% (v/v) FBS and 1% (v/v) penicillin/streptomycin in a humidified incubator at 37°C. Once tumors were palpable (50 mm^3^), mice were dosed with either 0.9 wt % saline (vehicle control), R-state PolyhHb or T-state PolyhHb for 2 weeks daily via tail-vein injection. All doses were administered as a 20% top load of the initial blood volume. Animals were euthanized at the endpoint of each experiment using CO_2_ inhalation. Tumors, livers, kidneys and spleens were harvested. Half of each organ was formalin fixed, paraffin embedded and used for immunohistochemistry (IHC) staining. The other half of each organ was flash frozen in liquid nitrogen for RNA and protein lysate preparation. Tumor tissue was used to isolate RNA for qPCR to quantify various hypoxia-induced genes. 18S rRNA expression was used as a normalization control. Prior to formalin fixation, tumors were weighed to assess growth differences.

### IHC staining

Paraffin embedded tissue sections were dewaxed and hydrated. LSAB+ System (DAKO) was used for hypoxyprobe and CD31 IHC staining according to the manufacturer’s instructions. Images were taken with the Cytation 5 Imaging Multi-Mode Reader (Biotek, Winooski, VT) and image analysis was performed using ImageJ software. Images were first deconvoluted and a threshold was placed on the region of interest (tumor) to calculate a percent staining area.

### Reverse transcription (RT) and qPCR

Total RNA was extracted using TRI Reagent and the Direct-zol™ RNA Mini Prep Plus kit (Zymo Research, Irvine, CA) according to the manufacturer’s instructions. One microgram of total RNA was used for first-strand DNA synthesis with the iScript cDNA synthesis kit (Bio-Rad Laboratories, Hercules, CA). qPCR was performed using human specific primers for BNIP3 (FW: CTTCCATCTCTGCTGCTCTC, RV: GTAATCCACTAACGAACCAA), P4HA1 (FW:CCCTGAGACTGGAAAATTGACCACAGC, RV: GGGGTTCATACTGTCCTCCAACTCCA), PGK1 (FW:TGGACGTTAAAGGGAAGCGG, RV: GCTCATAAGGACTACCGACTTGG), LDHA (FW: ATCTTGACCTACGTGGCTTGGA, RV: CCATACAGGCACACTGGAATCTC), 18S (FW: GAGGATGAGGTGGAACGTGT, RV: AGAAGTGACGCAGCCCTCTA) and iTaq SYBR Green Universal Master Mix (Bio-Rad). The expression of each target mRNA relative to 18S rRNA was calculated based on the threshold cycle (Ct) calculation as 2^-Δ(ΔCt)^, where ΔCt = Ct_target_−Ct_18S_ and Δ(ΔCt) = ΔCt_test_−ΔCt_control_.

### Tissue iron histopathology

Paraffin embedded tissue sections were dewaxed and hydrated. Perls 3,3’-diaminobenzidine tetrahydrochloride (DAB) staining, for the detection of non-heme iron, was performed on liver, spleen and kidney as previously described [39]. All images were acquired using an Olympus IX71 inverted microscope equipped with an Olympus DP70 digital camera.

### Western blot analysis

Frozen tissue samples were homogenized in the presence of ice-cold modified RIPA Buffer (50 mM Tris, 150 mM NaCl, 1% IgePal-630, 0.5% deoxycholate, 1 mM EDTA) containing protease inhibitors (Cocktail Set III, Calbiochem, CA). Lysates were centrifuged at 10,000 rpm at 4°C for 30 minutes. Supernatants were collected, divided into aliquots, and stored at -80°C. Protein concentrations were measured using the BCA protein assay (Thermo Scientific, Rockford, IL). Tissue lysates were resolved on 4–12% Bis-Tris gels, transferred to PVDF membranes, and blocked for 1 hr in TBS-T with 5% nonfat dry milk. Membranes were incubated overnight at 4°C with antibodies to HO-1 (#ab65080, dilution 1:2500) (Abcam, Cambridge, MA) and ferritin heavy chain (SPA 994, dilution, 1:2000) (Assay designs (Enzo), Farmingdale, NY) in TBS-T with 1% nonfat dry milk, washed, and then incubated with a relevant HRP-conjugated secondary antibody for 1 hr. Signal was developed using the ECL Plus kit and detected on HyperECL film. Densitometry analysis was performed using the ImageJ software (National Institutes of Health, Bethesda, MD) with normalization to beta-actin.

### Statistical analysis

Data are represented as mean +/- SEM. Tissues (n = 3-4/group) were analyzed using a one way ANOVA with a Bonferroni’s correction for multiple comparisons. P<0.05 was considered to be statistically significant for between group comparisons. All statistical analysis was performed using GraphPad Prism version 6.1, San Diego, CA.

## Results and discussion

### PolyhHb viscosity

**Fig 3:**
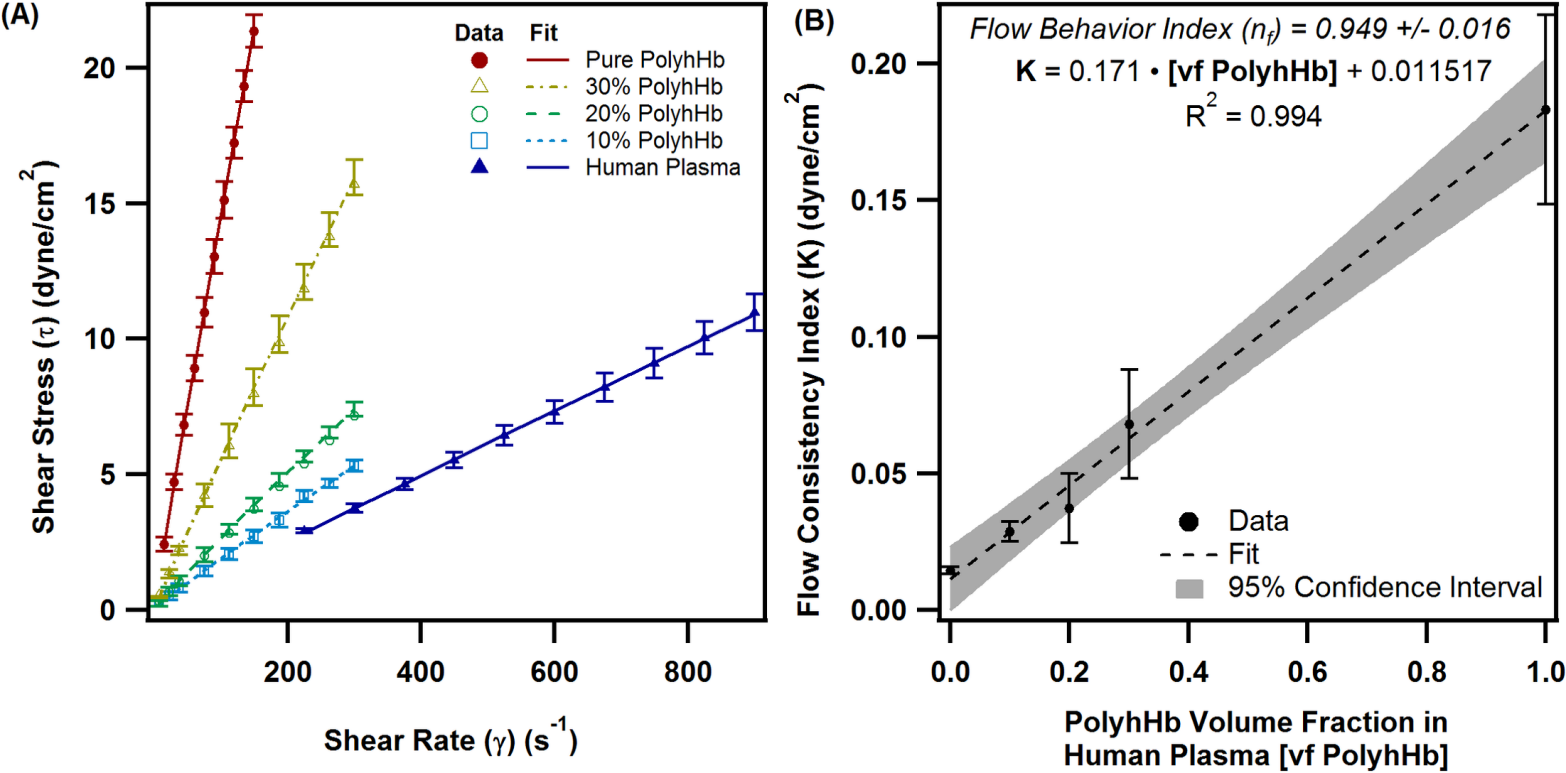
PolyhHb enhanced plasma viscosity and flow indices. (A) The shear stress versus shear rate plot for human plasma, pure polyhHb, and 10–30% mixtures of PolyhHb in human plasma by volume at 37°C. (B) The flow consistency index as a function of the volume percent of PolyhHb in human plasma. The error bars here depict the standard deviation (n = 3).

Fig 3A shows the rheological analysis of plasma PolyhHb mixtures. As the PolyhHb volume fraction (and thus concentration) increases, the viscosity of the mixture also increases. In addition, we observed that the viscosity of pure PolyhHb is approximately 10 times the viscosity of pure plasma. We also observed that the flow consistency index varied linearly with the volume fraction of PolyhHb in human plasma. All fluids demonstrated slight dilatant fluid behavior. However, there was no statistically significant effect of PolyhHb volume fraction on the flow behavior index. Additionally in Fig 3B, the flow behavior index (n_f_) approached Newtonian fluid behavior (n_f_ ≅ 0.95 ± 0.016). The flow behavior index was not plotted because the effect of PolyhHb on the flow behavior index was not statistically significant (p = 0.45). The flow behavior index is within the margin of error of the rheometer and may indicate that the PolyhHbs are Newtonian. However, to match the experimental data, the power law fit with a flow consistency index determined by the volume fraction of PolyhHb in plasma was used in the KTC model.

### Computational fluid velocity profiles

**Fig 4:**
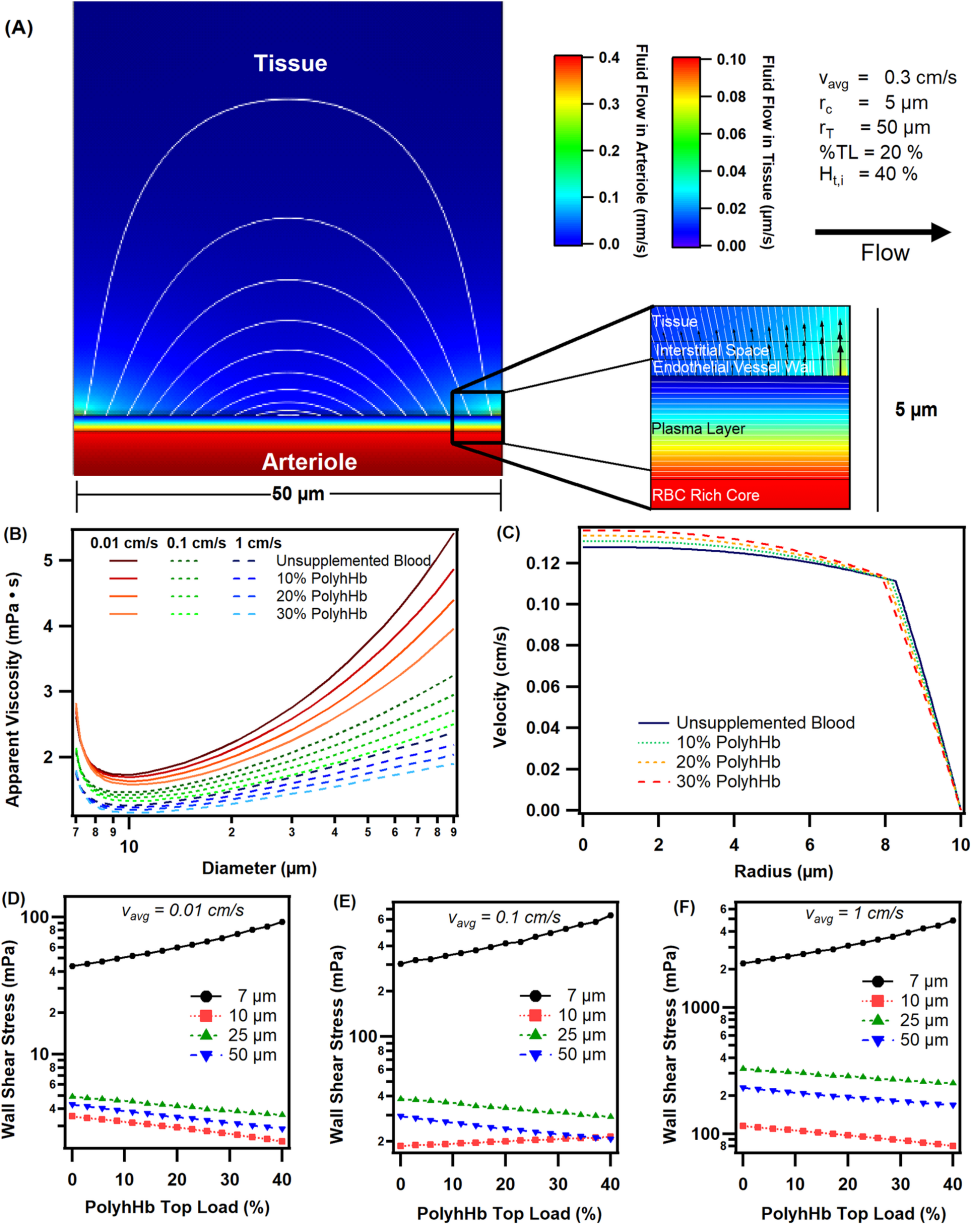
Velocity profiles, apparent viscosity and wall shear stress. (A) Velocity gradients represented by a color map through a 10 μm diameter arteriole and a 50 μm thick tissue space with an inlet average velocity of 0.3 mm/s. Note there are two color gradients for flow in the arteriole and flow in the tissue space. The white lines in the tissue space denote the flow paths for interstitial flow. (B) Apparent viscosities at varying diameters and flow rates with increasing top load adminstration of PolyhHb. (C) Radial velocity profile in the arteriole for a 10 μm diameter arteriole with v_avg_ = 0.1 cm/s. Wall shear stress calculated for varying top load transfusion percentages from 0 to 40% with PolyhHb at (D) 0.01 cm/s, (E) 0.1 cm/s and (F) 1 cm/s for arteriole diameters of 7, 10, 25, and 50 μm and a hematocrit of 40%.

Fig 4A shows the fluid velocity profile for an arteriole segment with a 10 μm diameter, 50 μm thick tissue space, and an average blood velocity of 0.03 cm/s. Flow in the arteriole is displayed on the mm/s scale, whereas flow in the tissue is displayed in the μm/s range. Streamlines have also been included to show the Starling flow behavior through the tissue space. As expected, the flow through the arteriole lumen is 1000 times greater than flow in the tissue space. In Fig 4B, we show the apparent viscosity calculated using the Hagen-Pouiselle equation for a range of blood vessel diameters, average velocities, and increasing top load transfusion of PolyhHb. Overall, increased transfusion of PolyhHb resulted in reduced apparent viscosity in the arteriole. Furthermore, we have identified a local minimum viscosity at an arteriole diameter close to 10 μm, which is comparable to previous blood flow models [44,45]. In Fig 4C, we show the radial velocity profile in the arteriole at a hematocrit of 45%. In the RBC rich core region (< 8 μm) velocity blunting was observed. As the amount of PolyhHb transfused increased, the resulting hemodilution led to increased parabolic behavior in the RBC core layer. In the RBC free region, a linear velocity profile was observed. Similar profiles were observed experimentally with a polymerized human serum albumin solution [47]. Motivated by the varying apparent viscosity of blood and PolyhHb in the arterioles, we decided to explore the effect of varying top load administration on the calculated average wall shear stress on the arteriole wall. The results of this analysis are shown in Fig 4D–4F. Interestingly, we found that increasing the transfusion volume of PolyhHb lead to increased wall shear stress at very small blood vessel diameters (7 μm). At moderately small blood vessel diameters (10 μm) increasing top load administration of PolyhHb decreased the wall shear stress for very low (0.01 cm/s) and very high (1 cm/s) average blood velocities. At moderate blood velocities (0.1 cm/s), we found that increasing the PolyhHb top load increased wall shear stress. As expected at moderately large (25 μm) and large (50 μm) blood vessel diameters increasing PolyhHb top load transfusion decreased wall shear stress. As anticipated from the apparent viscosity, large blood vessels exhibited slightly lower wall shear stress compared to moderately large vessels.

The results from our computational model indicate that top-load transfusion of PolyhHb may alter hemodynamics within tumor blood vessels. Despite PolyhHb solutions having a viscosity 10× greater than plasma, transfusion of PolyhHb decreased the apparent viscosity in the arteriole due to hemodilution. This is likely because reducing the hematocrit led to an increased cell-free plasma layer thickness and increased RBC volume fractions in the RBC rich core. These factors likely had a greater effect compared to increasing the plasma viscosity with PolyhHb. However, the effect of wall shear stress changed at small blood vessel diameters. This likely is a result of variations in the RBC free plasma layer and velocity profile blunting that occurs within the simulated arteriole [47,48]. Previous studies have reported that shear stress is a modulator of angiogenesis [49,50]. Therefore, increasing luminal shear stress can increase the formation of endothelial sprouts. This may be partially confirmed by the apparent reduction of angiogenesis demonstrated in the animal study presented in this work via the reduced vascular density. However, angiogenesis is also modulated by hypoxia mediated VEGF production [51]. Therefore, it is impossible to fully separate the individual contributions to angiogenesis given the complex biology.

### Arteriole inlet mixture O_2_ equilibrium

**Fig 5:**
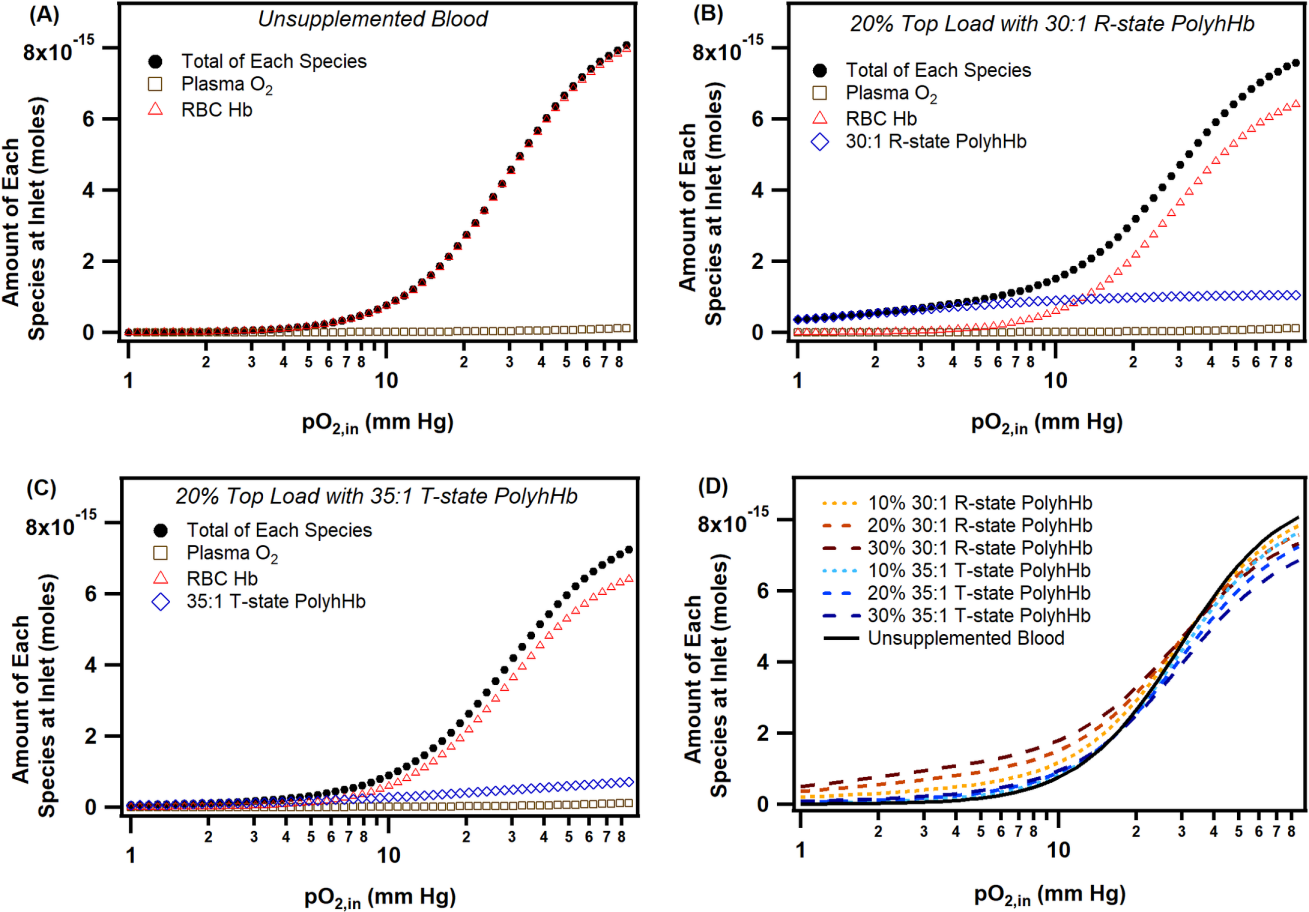
Amount of each oxygenated species at the arteriole inlet as a function of inlet O_2_ tension. These figures show the amount of each species (dissolved O_2_ in plasma, oxygenated Hb inside RBCs, oxygenated 35:1 T-/ 30:1 R-state PolyhHb) and total species as a function of the inlet O_2_ tension in the arteriole. (A) shows the equilibrium profile for unsupplemented blood, (B) shows the equilibrium profile for a 20% 30:1 R-state PolyhHb top load transfusion, (C) shows the equilibrium profile for a 20% 35:1 T-state PolyhHb top load transfusion, and (D) shows the total amount of O_2_ available from each O_2_ containing species at the inlet of the arteriole for varying levels of top load transfusions.

To understand how much of each oxygenated species exists in equilibrium at the inlet to the arteriole, we calculated the quantity of each species using the Hill equation and total Hb/PolyhHb concentration at varying pO_2,in_. The results from this analysis are shown in Fig 5. In general, Hb contained inside the RBCs carry the majority of O_2_ in the blood vessel at normoxic pO_2,in_ (> 10 mm Hg). In general, plasma caries very little dissolved O_2_ irrespective of either the pO_2,in_ or the type of PolyhHb transfused. Surprisingly, the 30:1 R-state had much more O_2_ available than the 35:1 T-state PolyhHb at a pO_2,in_ of 10 mm Hg, despite having a much lower P_50_. This results from the low O_2_ affinity of the 35:1 T-state PolyhHb. At atmospheric O_2_ tensions, the 35:1 T-state PolyhHb is only 70% saturated with O_2_ and thus has significantly less O_2_ available at all physiological pO_2,in_. Both the 35:1 T-state and 30:1 R-state PolyhHb demonstrate relatively constant changes in O_2_ saturation as a function of the pO_2_ compared to the Hb encapsulated inside RBCs.

At high inlet pO_2,in_ the majority of O_2_ is bound to Hb inside RBCs. This is because Hb inside RBCs constitute 70 to 90% of the total Hb available in the blood. As expected, the majority of O_2_ is found bound to various Hb species. T-state PolyhHb has a much lower O_2_ carrying capacity than either RBC derived Hb or R-state PolyhHb because of its reduced O_2_ carrying capacity when in the absence of pure O_2_. This indicates that T-state PolyhHb transports less total O_2_ than either RBCs or R-state PolyhHb under physiological O_2_ tensions.

### Overall O_2_ transfer rates

**Fig 6:**
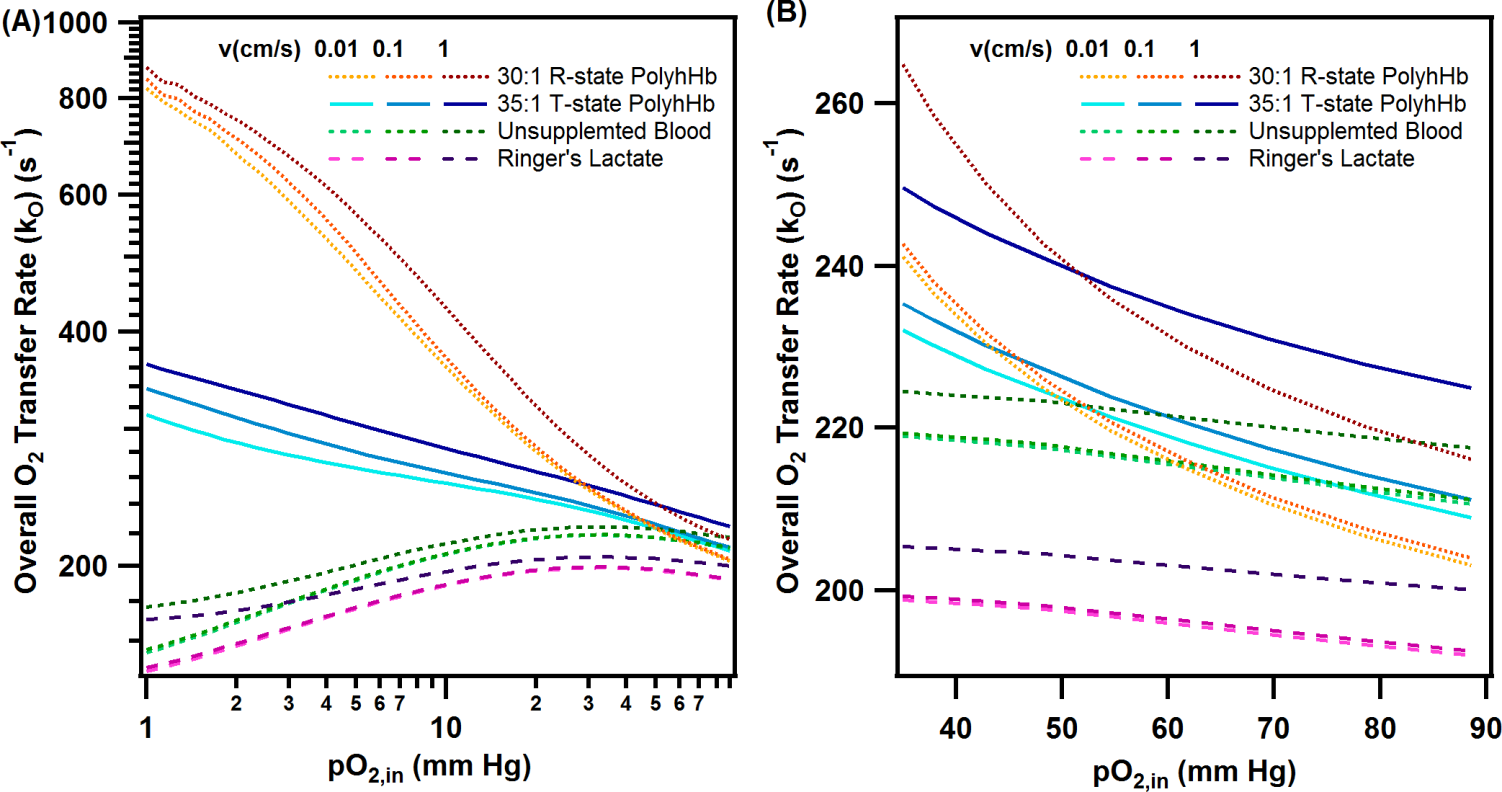
Overall O_2_ transfer rate as a function of inlet O_2_ tension at varying volumetric flow rate. This figure shows the overall O_2_ transfer rate calculated for 30:1 R-state PolyhHb, 35:1 T-state PolyhHb, unsupplemted blood and Ringer’s lactate solution for (A) all inlet pO_2,in_ (1–90 mm Hg) and (B) for normoxic pO_2,in_ (40–90 mm Hg). For all simulations the percent top load was set to 20% and the maximum rate of O_2_ consumption was set to 50 μM/s.

To understand the effect of varying PolyhHb transfusion on the O_2_ delivered to tumor tissue, we observed how the overall O_2_ transfer rate (k_o_) varied with 20% top load transfusion at different pO_2,in_. The overall O_2_ transfer rate for 20% top load transfusion of 35:1 T-state PolyhHb and 30:1 R-state PolyhHb at varying average blood velocity is plotted against pO_2,in_ in Fig 6. In general, all species behave similarly at normoxic pO_2,in_ (40–90 mm Hg) and low average blood velocities. As anticipated, the 35:1 T-state top load transfusion was computed to have slightly higher k_o_ compared to both the 30:1 R-state top load transfusion and unsupplemented blood. In addition, hemodilution via the simulated Ringer’s lactate transfusion led to a decrease in k_o_ for all pO_2,in_. In general, increasing average blood velocity leads to an increase in k_o_. Transfusion of R-state PolyhHb led to similar behavior to Ringer’s lactate diluted blood at high pO_2,in_. As anticipated from the O_2_ equilibrium curves of the 35:1 T-state and 30:1 R-state PolyhHb, at decreasing pO_2,in_ (< 40 mm Hg) transfusion of both the 35:1 T-state and 30:1 R-state PolyhHb led to increased O_2_ transfer into the tumor tissue. The change in k_o_ from low (0.01 cm/s) to moderate (0.1 cm/s) average fluid velocities is much less than the change in k_o_ from the moderate to high (1 cm/s) average fluid velocities for non-supplemented blood and the Ringer’s lactate simulations. In contrast, a moderate change from low to high arteriole average fluid velocities had a noticeable effect for both 35:1 T-state and 30:1 R-state PolyhHb.

The relative increase in the observed O_2_ flux likely results from the presence of the O_2_ carrying PolyhHb in the plasma layer. This makes the effective concentration of O_2_ in the plasma layer much higher than the concentration of plasma in unsupplemented blood. Under normoxic conditions, equilibrium mediated rate of O_2_ release by 30:1 R-state PolyhHb is much less than both the equilibrium mediated O_2_ release of T-state PolyhHb and normal blood. This reduced O_2_ offloading rate from the R-state PolyhHb is likely why the flux for R-state PolyhHb is less than both T-state PolyhHb and RBCs under normoxic conditions.

### O_2_ consumption rate

**Fig 7:**
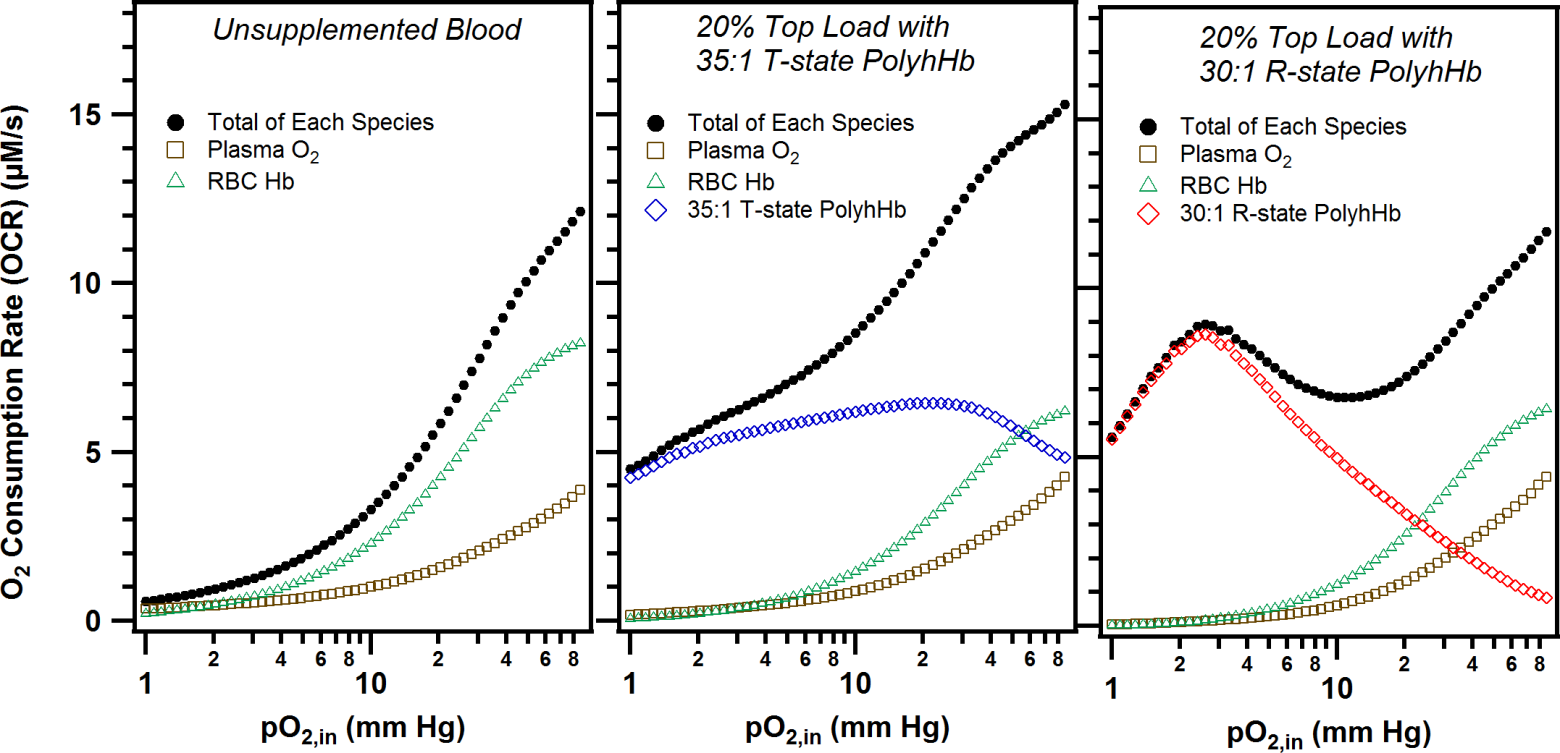
O_2_ consumption rate (OCR) as a function of inlet O_2_ tension. The OCR and individual contributions resulting from the various O_2_ carrying species in solution (i.e. dissolved O_2_ in plasma, oxygenated Hb inside RBCs, and oxygenated 35:1 T-/ 30:1 R-state PolyhHb) as a function of inlet O_2_ tension. OCR profiles are shown for (A) unsupplemented blood, (B) 20% blood volume top load of 30:1 R-state PolyhHb, (C) 20% blood volume top load of 35:1 T-state PolyhHb. For each of these simulations, v_avg_ = 0.03 cm/s, V_max_ = 40 μM/s, HCT = 40% and t_tissue_ = 50 μm.

We varied the type and amount of PolyhHb delivered over a spectrum of pO_2,in_ values to understand mass transport of O_2_, characterized by the O_2_ consumption rate (OCR), from the various O_2_ carrying species in the arteriole. Fig 7 shows the OCR for varying top load doses of PolyhHb compared to unsupplemented blood in a 10 μm diameter arteriole with an average fluid velocity of 0.03 cm/s, V_max_ of 40 μM/s, and 50 μm tissue thickness. Also included in parts A-C of this figure is a breakdown on the source of OCR from either RBCs, plasma or PolyhHb. As anticipated, RBCs deliver significantly more O_2_ at normoxic pO_2,in_s compared to the other species. However, at 40 mm Hg, the amount of O_2_ released by the 35:1 T-state PolyhHb exceeds O_2_ release by the RBCs. A similar effect occurs for 30:1 R-state PolyhHb at 30 mm Hg. As expected from the OEC profile, addition of 35:1 T-state PolyhHb leads to a significant increase in the OCR at all pO_2,in_. Furthermore, the increase in O_2_ release for the 35:1 T-state PolyhHb supplemented solutions increased with increasing doses of T-state PolyhHb. In contrast, addition of the 30:1 R-state PolyhHb led to increased O_2_ offloading at low pO_2,in_ but did not significantly affect the OCR compared to non-supplemented blood at normoxic pO_2,in_.

**Fig 8:**
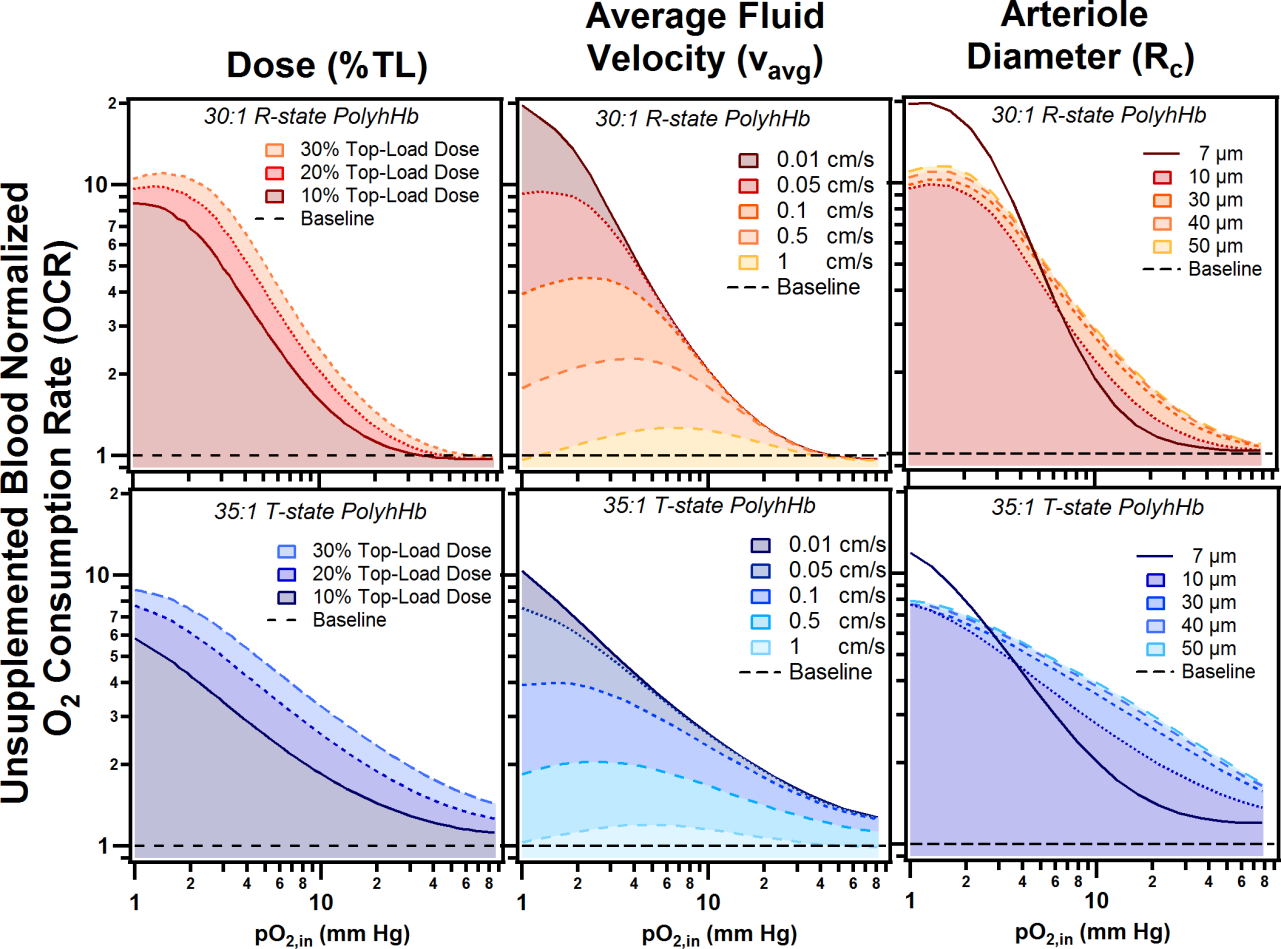
Unsupplemented blood normalized O_2_ consumption rate. This figure shows the O_2_ consumption rate normalized against unsupplemented blood (horizontal dashed line) for 30:1 R-state PolyhHb (top) and 35:1 T-state PolyhHb (bottom). For each species, the dose (left column), average blood velocity through the arteriole (middle collumn) and the arteriole diameter (right column) were varied. Other parameters were maintained as follows: (V_max_ = 40 μM/s, v_avg_ = 0.03 cm/s, D = 10 μm, and t_tissue_ = 50 μm).

We then varied several of the model parameters to examine how 35:1 T-state and 30:1 R-state PolyhHb transfusion can alter O_2_ mass transport. We examined the effect of varying the top load dose, average inlet velocity in the arteriole, and the diameter of the arteriole segment. The results of this analysis are shown in Fig 8. We found that O_2_ release for 35:1 T-state PolyhHb supplemented solutions increased with increasing doses of T-state PolyhHb. In contrast, addition of 30:1 R-state PolyhHb led to increased O_2_ offloading at low pO_2,in_ but did not significantly affect the OCR compared to non-supplemented blood at normoxic pO_2,in_. Interestingly, we found that at low volumetric flow rates, 35:1 T-state PolyhHb delivered consistently more O_2_ than unsupplemented blood below a pO_2,in_ of 20 mm Hg. As the volumetric flow rate increases, 30:1 R-state and 35:1 T-state PolyhHb hypoxia modulated increased O_2_ offloading approaches baseline conditions. For the 30:1 R-state PolyhHb, asymptotic behavior was observed as the average blood velocity increased. For all average blood velocities at normoxic pO_2,in_ (> 40 mm Hg), the amount of O_2_ offloaded was unchanged. As the average blood velocity increases, the amount of O_2_ offloaded diverges at increasing pO_2,in_. We suspect that the 35:1 T-state PolyhHb would have similar behavior to the 30:1 R-state at very high pO_2,in_ (> 90 mm Hg). However, this range is not physiologically relevant for non-hyperbaric type analysis. In our analysis, we found that increased blood vessel diameter lead to increased O_2_ delivery at all pO_2,in_. This effect is constant for 30:1 R-state PolyhHb; whereas the OCR modifying effect is asymptotic at very low pO_2,in_ (< 3 mm Hg) for 35:1 T-state PolyhHb. Interestingly, when the arteriole collapses down to the capillary scale, the OCR drastically increased for both PolyhHb species under hypoxic conditions.

The results of the simulations performed here contrast with some of the results presented in previous simulations investigating HBOCs for solid tumor oxygenation [25]. While O_2_ delivery was dependent on the P_50_ of the 30:1 R-state and 35:1 T-state PolyhHb, increasing blood flow rates appears to mask this effect. For example, the increase in O_2_ delivery at high blood v_avg_ (1 cm/s) the 30:1 R-state PolyhHb behaves similarly to the 35:1 T-state PolyhHb. In addition, T-state PolyhHb was found to boost oxygenation at all pO_2.in_ and not only at its respective P_50_. We also found that R-state PolyhHb did not limit O_2_ transport as stated in the Gundersen and Palmer simulation [25]. We believe these differences reflect the addition of the more physiologically relevant blunted velocity profile after applying the Quemeda constitutive equation in the current study.

**Fig 9:**
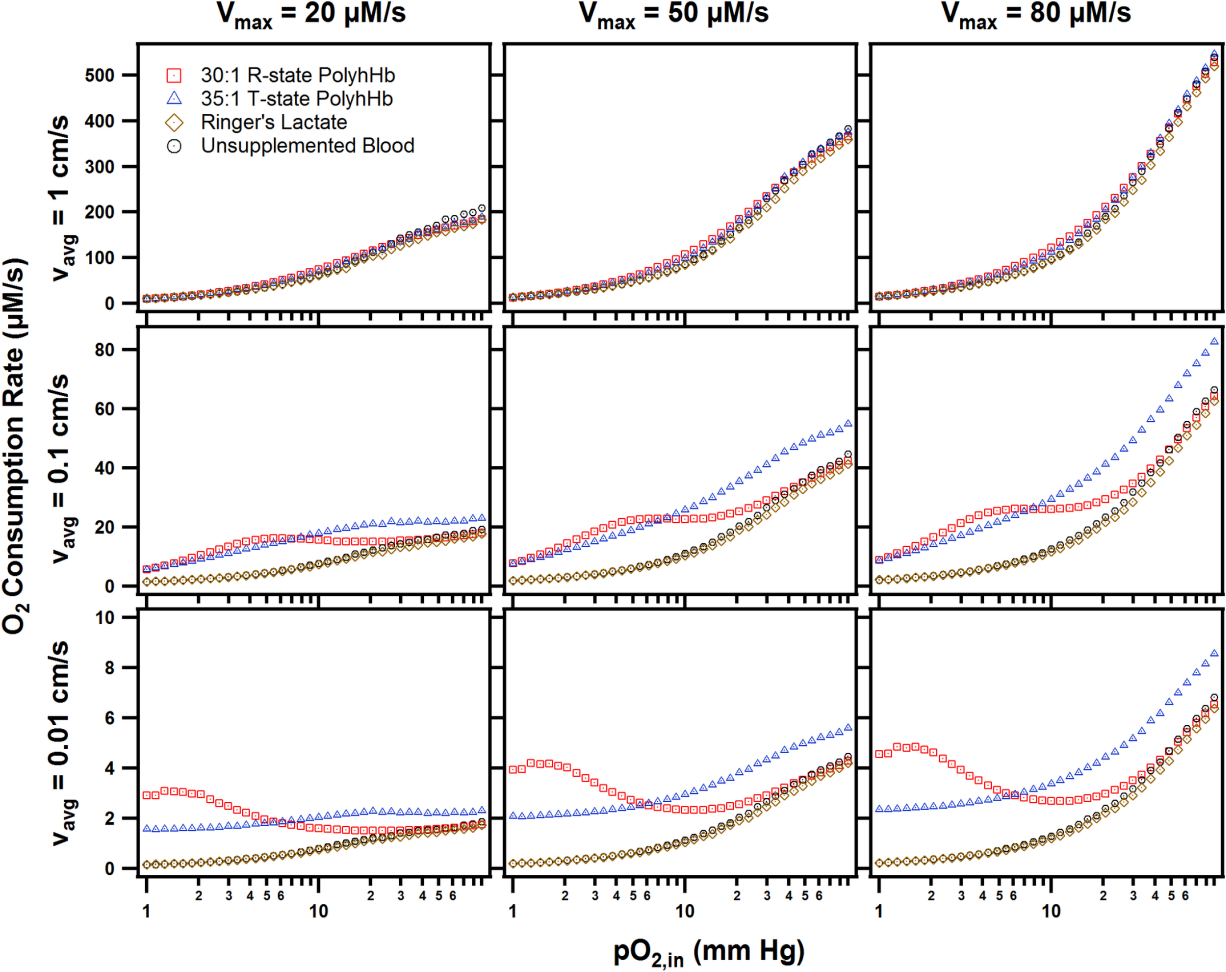
OCR as a function of inlet O_2_ tension, average inlet blood velocity, and maximum rate of O_2_ consumption. These figures show the OCR for 30:1 R-state PolyhHb, 35:1 T-state PolyhHb, and unsupplemented blood. The average inlet blood velocity was varied from 0.01 to 1 cm/s (rows). The maximum rate of O_2_ consumption was varied from 20 to 80 μM/s (columns). For all PolyhHb simulations, the top-load was set to 20% of the blood volume. (t_tissue_ = 50 μm.).

We also explored the effect of simultaneously varying the maximum rate of tissue O_2_ consumption (V_max_) and average arteriole blood velocity (v_avg_) on the OCR. The results from this analysis are shown in Fig 9. The increased V_max_ also decreases the enhanced OCR in the hypoxic regions compared to non-supplemented blood. In this model, the fluid velocity had an overall greater effect on increasing O_2_ mass transport into the tissue compared to increasing O_2_ consumption by cells in the tissue space. This motivated us to explore the relative effects of varying environmental and dosage parameters in our computational model shown in the subsequent section.

### Model sensitivity

**Fig 10:**
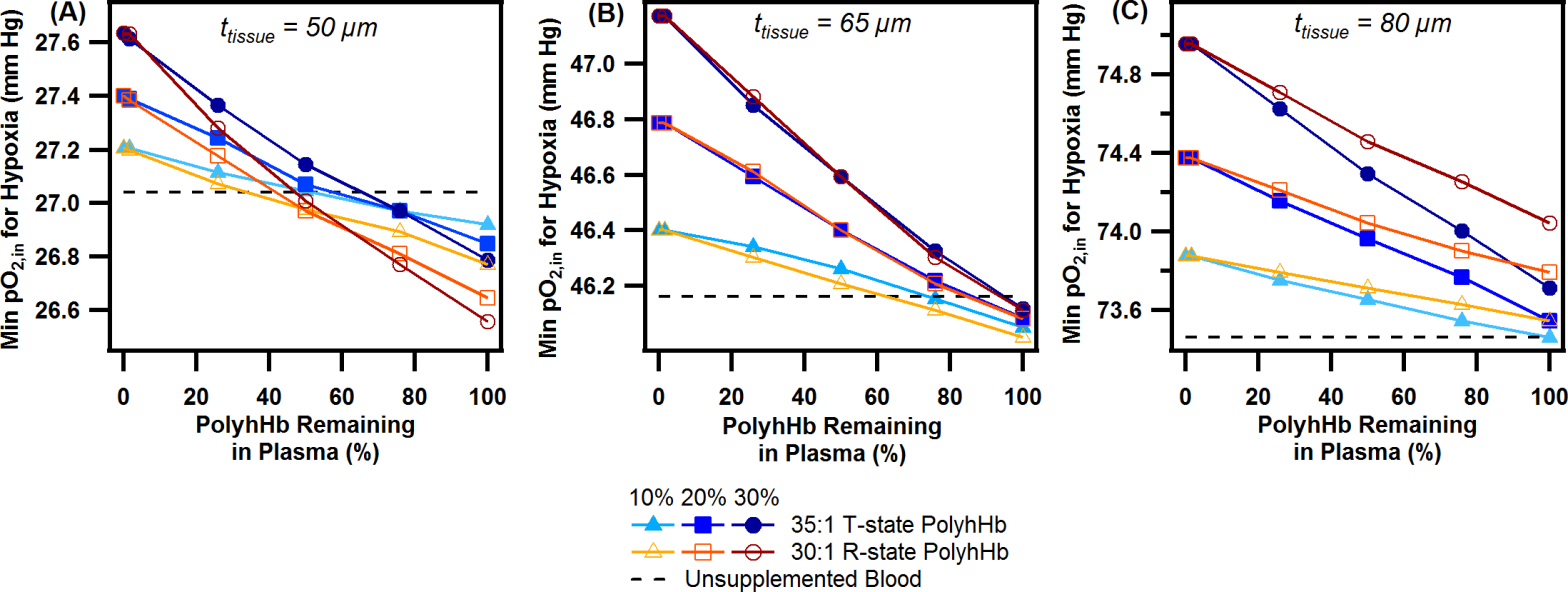
False detection rate (FDR) sensitivity to variable model parameters for k_0_ and OCR. These figures depict the FDR for (A) k_0_ and (B) OCR for 35:1 T-state PolyhHb, 30:1 R-state PolyhHb, and unsupplemented blood at varying pO_2,in_ values. Below each column depicts if the effect of increasing the parameter is positive (+), negative (-), or mixed (m) for the T-state PolyhHb, R-state PolyhHb, and non-supplemented blood.

Because the scope of the KTC model is limited by the required assumptions and the limited phase space of the chosen simulation parameters, we investigated the effects of varying simulation parameters on the OCR and k_o_ by comparing the resulting false detection rate (FDR) log of each of the selected parameters. The results of this analysis are shown in Fig 10. The FDR analysis indicated which model parameters have the greatest effect on the OCR and k_0_. If the FDR for a model parameter is greater, the effect of changing that model parameter will lead to a greater change in the OCR or k_0_ compared to changing other parameters with lower FDR values. Note that each FDR test was performed for varying pO_2,in_ and type of PolyhHb. The FDR analysis correctly predicts that the %TL and C_PolyhHb_ has no effect on the mode (FDR Log Worth ≪ 1 × 10^−6^). For the sensitivity analysis, these parameters could vary at the same levels as in the PolyhHb supplemented simulations. FDR analysis alone was insufficient to determine if the effect was positive or negative. To estimate the sign of these effects, we observed the trends of each varying parameter set for all pO_2,in_ and estimated if the effect was overall positive, negative, or mixed. If an event resulted in a positive effect, this meant that increasing that parameter led to an increase in the resulting k_0_ and OCR. In contrast, a negative effect indicated that increasing the parameter led to a decrease in the resulting k_0_ and OCR. A mixed effect indicated that increasing the parameter had some combination of positive and negative effects.

Overall, the radius of the arteriole has the greatest relative effect on k_0_. This is expected because of the restrictions of this simulation. Note that we have restrained the tissue thickness while varying the radius of the arteriole. Therefore, the same volume of cells required O_2_ delivery at a relatively constant rate despite the smaller blood vessel surface area, requiring a greater O_2_ flux across the blood vessel wall. The inlet average velocity appears to have less of an effect for 35:1 T-state PolyhHb. As a result, we speculate that 35:1 T-state PolyhHb has a relatively constant rate of O_2_ offloading. Increasing the rate of O_2_ consumption in the tissue space led to an increase in O_2_ flux for both PolyhHbs, but a decrease for unsupplemented blood. We suspect this occurs with PolyhHb in the plasma free layer and is a result of non-cooperative O_2_ offloading, a similar effect was observed for increasing K_M._

For 30:1 R-state PolyhHb, increasing HCT lead to increased OCR at very low pO_2,in_ and decreased OCR at high pO_2,in_. This may indicate that R-state PolyhHb provides more O_2_ at lower hematocrit levels. As expected, with respect to the flow rate dependence on O_2_ mass transfer, the fluid velocity had the greatest effect on the OCR. The effects were further explored in a previous section (Fig 9). Changing the V_max_ and the K_M_ resulted in similar effects for both PolyhHb transfusions and non-supplemented blood. It appears that the 35:1 T-state PolyhHb is much more sensitive to reduction in the blood vessel radius compared to 30:1 R-state PolyhHb and unsupplemented blood. The OCR is more sensitive to the 35:1 T-state PolyhHb concentration, but not the volume of the dose. However, for both the k_0_ and OCR FDR results, the environmental conditions including the OCR of the cells and the arteriole geometry have a greater effect than varying the volume or concentration of PolyhHb.

### Computed effect of PolyhHb on hypoxia

**Fig 11:**
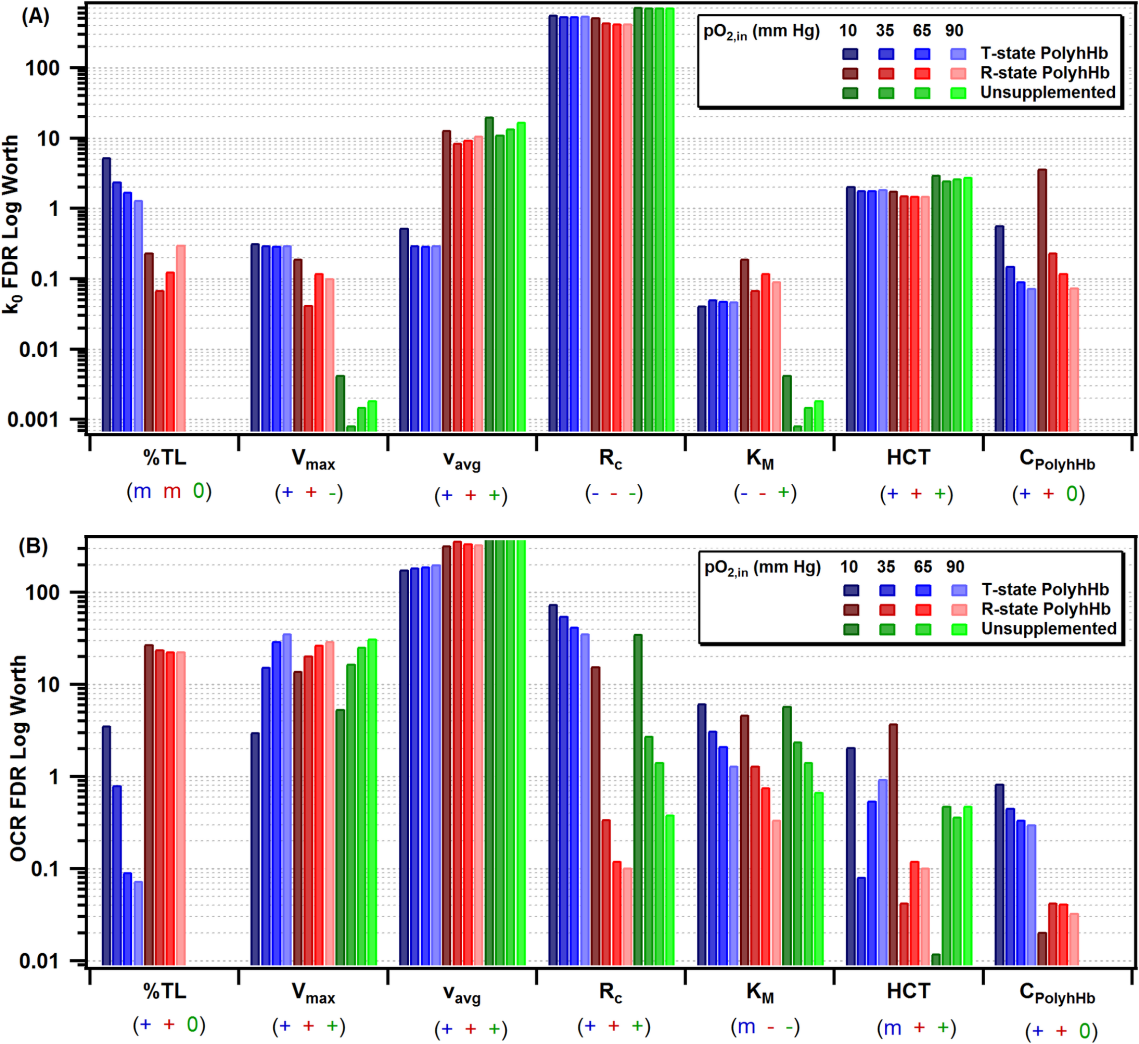
Minimum inlet O_2_ tension as a function of the amount of PolyhHb remaining in the plasma at varying tissue layer thicknesses. The figures show the minimum inlet O_2_ tension that is observed before a hypoxic (< 5 mm Hg) region is observed. The thickness of the tissue region was varied from (A) 50 μm, (B) 65 μm, and (C) 80 μm. The amount of PolyhHb transfused was varied from 10 to 30% top load. The percentage of initially administered PolyhHb remaining in the circulation was decreased. Unsupplemented blood was used as a control. For this simulation, the hematocrit was set to 0.45, and the flow rate was set to 0.03 cm/s.

To approximate the effect of PolyhHb top load transfusion and clearance on the resulting hypoxic volume, we examined the minimum pO_2,in_ before hypoxic behavior was observed against the PolyhHb remaining in the plasma for various top load transfusion percentages, type of PolyhHb, and tissue thicknesses. The results of these simulations are depicted in Fig 11. We observed that PolyhHb was more effective for small tissue layer thicknesses (50 μm). 30:1 R-state PolyhHb demonstrated improved behavior compared to 35:1 T-state PolyhHb at these small blood vessel diameters. In addition, at this particular blood vessel diameter, lower doses of PolyhHb began to restore normoxia to greater extent than higher doses. This behavior tends to occur when a moderate amount of PolyhHb remains in circulation (50–80% of the original dose). As expected the 35:1 T-state PolyhHb reduced relative O_2_ delivery compared to the 30:1 R-state PolyhHb at low pO_2,in_, while R-state PolyhHb maintained a more pronounced attenuation of hypoxia, despite a lower concentration in the plasma. Also as anticipated, when PolyhHb was depleted of O_2_, the threshold for hypoxia increased and the magnitude of this effect increased with increasing doses of PolyhHb solution. At a moderate tissue thickness (65 μm), 35:1 T-state and 30:1 R-state PolyhHbs were only effective when the majority of the initial dosage was present in the circulation (80–100%). Unlike the system with a tissue thickness of 50 μm, both 35:1 T-state and 30:1 R-state PolyhHbs exhibited similar effectiveness at reducing hypoxia. At large tissue thicknesses (80 μm), no PolyhHb formulation or dose was effective at reducing the effect of hypoxia. Furthermore,30:1 R-state PolyhHb performed significantly worse than 35:1 T-state PolyhHb.

The increase in hypoxia due to the decreasing vascular density is consistent with microcirculatory oxygenation observed in intravascular microscopy studies [52]. This also indicates that the architecture of the microcirculatory system in the tumor environment continues to be a strong effector on HBOC mediated oxygenation of tumor tissue.

### Animal studies

**Fig 12:**
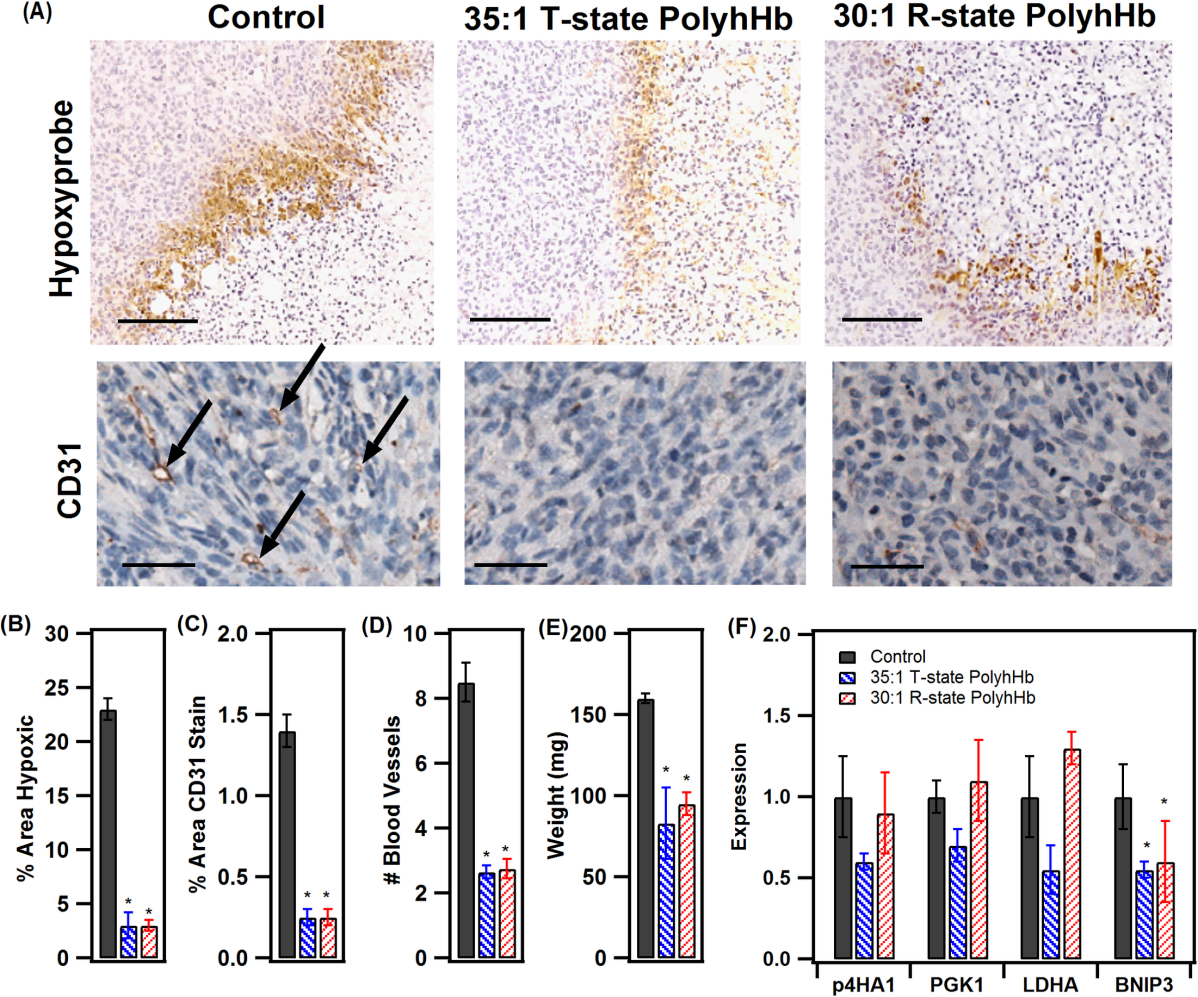
Biomarkers for tumor growth and oxygenation. (A) Immunohistochemical staining using antibodies specific for the detection of hypoxic adducts (hypoxyprobe) (top panel) (scale bar = 200 μm) and CD31 (bottom panel) (scale bar = 50 μm) on tissue sections of MDA-MB-231 orthotopic breast tumors. Arrows indicate vessels in the CD31 stained images. (B-D) Images were deconvoluted and a threshold was created for each stain to determine percent hypoxic area (B) (mean ± SEM, n = 3–4 mice × 4 images), percent area of CD31 stain (C) (mean ± SEM, n = 3–4 mice × 4 images) or number of blood vessels (D) (mean ± SEM, n = 3–4 mice × 4 images). (E) Tumor weights at end of trial (mean ± SEM, n = 3–4). (F) Hypoxia-inducible gene expression in tumors treated with either the control (saline), T-state PolyhHb or R-state PolyhHb. mRNA levels of P4HA1, PGK1, LDHA and BNIP3 were analyzed in tumors established with MDA-MB-231 cells normalized to the control (mean ± SEM, n = 3–4 mice × 3 replicates). (* p < 0.05, ANOVA with post-hoc correction).

To explore the effectiveness of PolyhHb in an *in vivo* tumor model, we evaluated the differences in the extent of intra-tumoral hypoxia, blood vessel density, tumor growth, and the expression of hypoxia-inducible genes in mice treated daily for two weeks with a vehicle control versus R- or T- treated mice. Immunohistochemical staining of tumors derived from the orthotropic transplantation of human metastatic mammary carcinoma cells (MDA-MB-231 cells) into the mammary fat of NOD SCID gamma mice revealed enhanced hypoxyprobe staining in control tumors compared to those tumors from mice treated with either the 30:1 R-state or 35:1 T-state PolyhHb solution (Fig 12A, top panel). This suggests reduced levels of hypoxia in mice treated with both PolyhHbs. Quantifying the extent of hypoxia probe staining across the entire tumor cross section also showed a significant decrease in both the R-state and T-state PolyhHb treated groups (Fig 12B). Tumor sections were also stained with an anti-CD31 antibody to detect endothelial cells that form blood vessels within each tumor followed by image analysis to detect the level of angiogenesis by measuring the area covered by the CD31 stain (Fig 12C) as well as by counting the number of blood vessels in each tumor (Fig 12D). These results display a significantly decreased number of blood vessels for tumors treated with either PolyhHb solution. We also measured the final tumor size by weighing the tumor mass at the endpoint of the experiment along with determining the cross-sectional area of each tumor section. These results shows that tumor growth was impaired in mice treated with PolyhHb, suggesting a slower proliferation rate and subsequent delay in tumor growth (Fig 12E). RNA isolated from these tumors were analyzed via qPCR for various hypoxia-inducible genes such as P4HA1, PGK1, LDHA and BNIP3 (Fig 12F). For all four genes, T-state PolyhHb treated tumors showed a 25%-50% decrease in expression as compared with the control tumors. However, the R-state PolyhHb treated tumors only showed a lower expression in BNIP3 compared to the control.

In our computational study, we found that T-state PolyhHb may not be fully oxygenated under normoxic conditions. This should indicate that T-state PolyhHb oxygenated tissues to a lesser extent compared to R-state PolyhHb. However, we observed no statistically significant differences in oxygenation between T- and R-state PolyhHb in the animal studies. We suspect that this is because a moderate transfusion load was selected for the study. If a larger volume of blood was removed and replaced with T-state PolyhHb, a significant reduction in the O_2_ carrying capacity of blood may have been observed. The modified Krogh tissue cylinder model described here does not fully describe the complex microvascular geometries in the tumor. As such, we were unable to replicate the hypoxic reduction observed in the animal model. However, we did observe that PolyhHbs were more effective at high vascular densities (i.e. represented by low tissue thicknesses). We expect that this scenario reflects the fully oxygenated tissue prior to initiation of tumor growth. Since the mice treated with R- and T- state PolyhHb had smaller tumors, it is likely that limited angiogenesis was required to maintain tumor growth. After transfusion of the PolyhHb solution, increased oxygenation led to a reduction in VEGF production. This in turn may have led to normalization of the tumor microcirculatory system. As more normalized vasculature was observed at the end of the animal study, we believe that PolyhHb transfusion resulted in a significant reduction in chronic hypoxia. Our sensitivity analysis via FDR analysis indicates that oxygenation is heavily dependent on the conditions of the tumor microenvironment. The radius of the capillary and blood flow rate were found to have a greater effect than either the dosage or the concentration of PolyhHb in the screened results. This is interesting given the transient and heterogeneous nature of tumor tissue. It is likely that PolyhHb may give drastically different responses depending on tumor growth and extent of vascularization even in the same type of tumor.

### Tumor and clearance organ tissue exposure to PolyhHb

**Fig 13:**
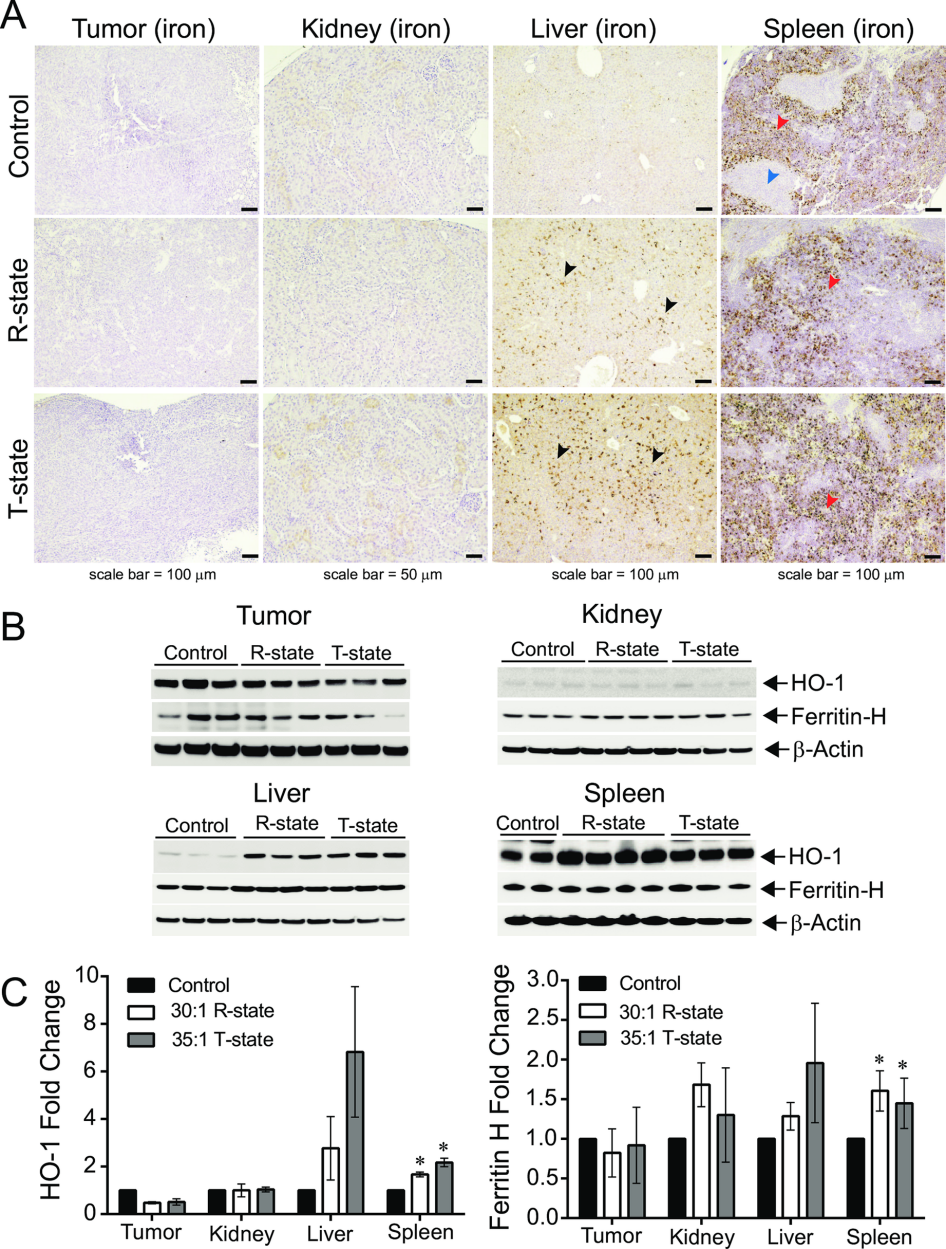
Tissue markers of iron accumulation following PolyhHb treatment. (A) Light microscopy images of representative Perls DAB stained tissue sections from control mice and mice after treatment with PolyhHb in the R- and T-states. Control, spleen shows the characteristic brown staining pattern of red pulp (red arrow) and white pulp (blue arrow). Tumor and kidney tissue are absent of iron accumulation with or without PolyhHb treatment, while the liver shows iron accumulation in macrophage and monocyte cells (black arrows). Expansion of red pulp regions in the spleen (red arrows) is observed following both R- and T-state PolyhHb treatment. Images were obtained at (100 and 200× magnification (objective 10×, 20×) and scale bars represent 100 μm for the tumor, liver and spleen and 50 μm for the kidney. (B) Western Blot analysis is shown for tumor, kidney, liver and spleen HO-1 and ferritin H protein expression. (C) Densitometry analysis of (B) showing significantly increased splenic HO-1 (P = 0.0329 *, T-state PolyhHb vs. control, n = 3–4) and (P = 0.0013*, R-state PolyhHb vs. control, (n = 3–4). An increase in ferritin H was also observed in the spleen following PolyhHb treatment versus the control (P = 0.0471*, n = 3–4), by ANOVA with a Multiple Comparisons Test.

We determined tissue exposure to PolyhHb by identification of iron, heme oxygenase-1 (HO-1) and ferritin heavy (H) chain in the tumor, spleen, liver and kidney at study termination (Fig 13). Visualization of iron positive regions within the Perls DAB stained tissue is shown in Fig 13A for tumor, kidney, liver and spleen following treatment with vehicle control or treatment with R- or T-state PolyhHb. The majority of iron is observed following PolyhHb dosing within resident macrophages in the liver (black arrows) and within the red pulp (red arrows) of the spleen. Spleen white pulp is designated by a blue arrow in control tissue and subsequent expansion of the red pulp is evident following PolyhHb infusion. The heme-metabolizing enzyme and iron storage protein, HO-1 and ferritin H, respectively were evaluated by Western Blotting as shown in Fig 13B. Tumor tissue from mice treated with R- or T- state PolyhHb showed reduced HO-1 and ferritin H compared to tumor tissue from mice treated with vehicle control only (Fig 13C). HO-1 and ferritin H staining in the kidney tissue did not differ from control following infusion of PolyhHb (Fig 13B and 13C), indicating minimal renal clearance in treated animals. Liver HO-1 and ferritin H were increased following PolyhHb treatment (Fig 13B and 13C), consistent with increased iron deposition observed in Perls DAB stained liver tissue. Predominant splenic clearance of PolyhHb is observed by significantly greater HO-1 induction following T-state (P = 0.0329) and R-state (P = 0.0013) PolyhHb treatment compared to non- treated control animals (Fig 13B and 13C). Similarly, ferritin H increased significantly following PolyhHb treatment (P = 0.0471) compared to non- treated control animals (Fig 13B and 13C).

Unfortunately, our computational model is not able to access toxicity, accumulation, and clearance for *in vivo* systems. These behaviors are vital to assess the safety and efficacy of any large volume O_2_ carrier transfusion. Tissue analysis results indicate that PolyhHb cleared primarily through the liver and spleen. This mode of clearance is especially important because renal clearance can result in severe kidney damage which may include oxidative tissue injury, tubular failure, and renal failure [53–55], suggesting the size of the engineered PolyhHb is sufficient to reduce the risk of acute renal injury [39]. We also observed that extravascular translocation of the PolyhHb into the tumor mass was negligible. This indicates that the PolyhHb no-flux boundary condition set at the vessel wall was valid for our simulation. We were unable to measure the circulatory PolyhHb during these trials. However, we can infer the pharmacokinetics and the circulatory half-lives given the results of a previously performed down-selection analysis of polymerized bovine Hbs [39]. We expect the circulatory half-lives of these materials to be between 20 and 30 hours in small animals.

### Retrospective analysis previous experimental studies

**Fig 14:**
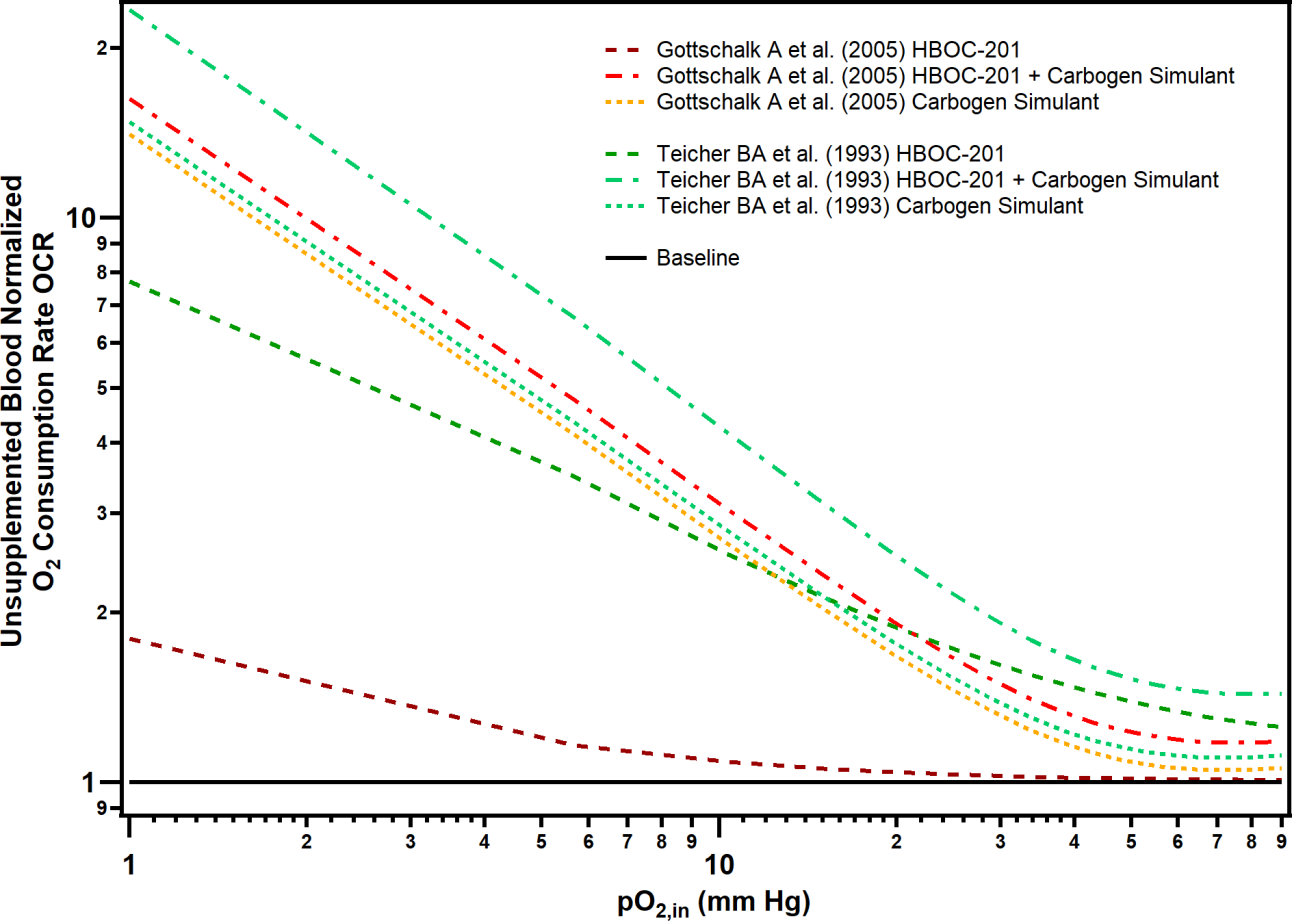
Retrospective analysis of expected increase in OCR from previous experimental models. This figure shows the OCR normalized against unsuplemented blood (horizontal solid line) compared to computational estimates of HBOC-201, carbogen gas, or HBOC-201/carbogen gas co-therapeutic as described in a 1993 study by Teicher et al. (9L Gliosarcoma) and a 2005 study by Gottschalk et al. (R1H Rhabdomyosarcoma). (V_max_ = 40 μM/s, v_avg_ = 0.03 cm/s, D = 10 μm, and t_tissue_ = 50 μm).

In addition to comparing our computational results to our *in vivo* results, we also retrospectively examined the data collected from a myriad of previous studies that used polymerized Hb to facilitate tumor oxygenation. For many of these studies, a commercial polymerized bovine Hb (HBOC-201 ®, Hemopure) was used; however, the P_50_ of this material (38 mm Hg) was not significantly different than the P_50_ of the 35:1 T-state PolyhHb (37.35 mm Hg) used in this current study [56]. Many of these previous studies also used carbogen gas (95% O_2_ 5% CO_2_) and/or hyperbaric O_2_ (100% O_2_) as a control. The use of carbogen gas and other parameters were simulated as described in S1 Text. The results of this retrospective computational analysis are shown in Fig 14. We found that the HBOC-201 dosage levels reported by Gottschalk et al. were likely not sufficient to increase O_2_ transport to the tumor tissue as evidenced by the slight deviation from baseline conditions. We were able to capture the dose modifying effects of the carbogen simulant. We also observed that T-state PolyhHb likely still provides more O_2_ under normoxic conditions. However, the carbogen simulant had a stronger effect under hypoxic conditions.

In the 1993 study by Teicher *et al*., the effect of HBOC-201® on tumor oxygenation in a rat 9L gliosarcoma and rat 13672 mammary adenocarcinoma was examined [57]. The 9L gliosarcoma was responsive to treatment with HBOC-201®. In our computational model, we observed an increased OCR when HBOC-201 was applied under normoxic conditions. However, carbogen outperformed HBOC-201 under hypoxic conditions. These computational results are comparable to the increase in tissue oxygenation observed in previously conducted animal trials [57]. A separate study reported that the 9L gliosarcoma is characterized by high vascularization, normal blood volume, and increased blood vessel diameter (10 μm– 20 μm) compared to normal tissue [58]. From our FDR effect analysis, we predict that a low O_2_ affinity HBOC would be more effective in oxygenating this type of tumor tissue under these microcirculatory conditions. The increase in oxygenation is confirmed from the results of Teicher *et al*.’s study. The 13672 mammary adenocarcinoma was also responsive to transfusion of HBOC-201®. However 24 hours after transfusion, the tissue became more hypoxic versus pre transfusion conditions for both air (median pO_2_: 12.3 → 5.3 mm Hg) and carbogen (median pO_2_: 37.3 → 12.3 mm Hg). Interestingly, similar effects were observed when a 100% O_2_ solution was transfused [59]. To our knowledge, no study has explored the microcirculatory system of 13672-mammarry adenocarcinomas. However, this hypoxic effect is confirmed for T-state PolyhHb in our model when the tissue is poorly vascularized. Notably, in both tumors, a combined regimen of carbogen and HBOC-201® was more effective than the use of HBOC alone. From our model, this behavior is expected for low O_2_ affinity HBOCs due to increasing the overall supply of O_2_. From our computational model, this may lead to a fully saturated low O_2_ affinity HBOC in the lungs and thus more O_2_ delivery to the tissues. A series of studies in 2005 explored transfusion of HBOC-201® on a R1H rhabdomyosarcoma in a WAG/Rij rat model [24,60]. Both of these studies found that HBOC-201 ® had no significant effect on tumor oxygenation. In general, rhabdomyosarcomas are characterized by lower blood flow [61]. Because of this, we would expect that a low O_2_ affinity PolyhHb will be more effective. However, these studies used very low concentrations of the HBOC-201 (4–6 mg/mL in plasma) compared to what was investigated in this current study (15–25 mg/mL in plasma) [24,60]. At these low HBOC concentrations, our computational model would suggest an increase in the OCR of less than 5% whereas increasing available O_2_ via carbogen would increase it by up to 20%. Furthermore, rhabdomyosarcoma’s are characterized by increased myoglobin which would make them suitable for carbogen based O_2_ delivery via elevated O_2_ storage in the tissue space [62]. These results were confirmed by our retrospective computational analysis wherein we found that the dosage level in these studies would have an insignificant effect on tissue oxygenation. We can also explain the increase in hypoxic behavior that was observed in the study by the PolyhHb clearance as shown in our analysis of computed hypoxia with clearance. A 1996 study by Nozue *et al*. examined the effect of various modified Hb species on tumor oxygenation in a HGL9 human glioma in nude mice and FSall mice fibrosarcoma in C3H mice [22]. This study showed that a high affinity O_2_ carrier, bovine-DIBS-CmHb, did not sufficiently oxygenate the tumor. Comparing this to our predictive model, we hypothesize that either the blood flow rate was too high, the degree of vascularization was inadequate, or the circulatory half-life was too low. Considering the low O_2_ affinity PolyhHb adequately oxygenated the tumor, we hypothesize that the low circulatory half-life may explain the poor oxygenation observed.

## Conclusions

In our computational tumor O_2_ transport model, we found that increasing the PolyhHb dose, residence time, or tumor-cell oxygen consumption increased O_2_ transport into the tissue space. For R-state PolyhHb, the effects were magnified at lower pO_2,in_. Given that blood flow in most tumors are characterized by low flow rates and low arteriole pO_2_s [63,64], both T- and R-state PolyhHb should facilitate increased oxygen delivery compared to non-supplemented blood. This was partially confirmed by the animal studies. We observed several biomarkers for increased oxygenation including decreased hypoxic volume, decreased angiogenesis, and decreased expression of hypoxia-inducible genes. However, it was not possible to quantify residence time of the various Hb species within the tumor in our animal model. This is especially interesting given that the initial total O_2_ capacity was the same. We also noted that the computed flux of O_2_, characterized by k_0_, increased for low pO_2,in_ for 30:1 R-state PolyhHb. This would imply that O_2_ was more evenly distributed within the tumor mass. However, the effect was always greater for 35:1 T-state PolyhHb, which indicates that T-state PolyhHb would lose O_2_ faster compared to R-state PolyhHb. Despite these inherent differences, both PolyhHbs resulted in similar oxygenation biomarkers in the animal model. We anticipate that these result from elevated O_2_ transfer from the RBCs to the PolyhHb in the plasma and the presence of O_2_ carriers in the cell free layer.

There have been many approaches to combat hypoxia in solid tumors. Many hypoxia targeting therapies that interfere with metabolic pathways were demonstrated in previous studies [65–68]. Unfortunately, these proposed therapeutics require targeted delivery to cancer cells and are thus limited by nonspecific distribution, unwanted elimination, multidrug resistance, intratumoral pressure and many of the other problems currently plaguing drug delivery [69]. In light of these concerns, some therapeutic modalities aim to increase the O_2_ supply to tumor tissue through strategies including hyperbaric and carbogen gas based approaches. While well suited for radiotherapy, these methods are temporary and can result in systemic oxidative stress under prolonged exposure [70]. An alternate method to temporarily increase tumor oxygenation consists of vascular normalization via inhibition of vascular endothelial growth factor receptor (VEGFR) by drugs such as Avastin (bevacizumab) or cediranib [71,72]. Unfortunately, the vascular normalization effect of VEGFR inhibitors is transient and eventually leads to over-pruning of the tumor vascular networks. The resulting lack of blood flow can starve the tumor, which leads to increased hypoxia in the tumor. Because of this, the FDA has declared that Avastin is neither safe nor effective for use in the treatment of breast cancer [73,74]. The declining use of antiangiogenic therapies in some cancers indicates that alternative means of increasing tumor oxygenation and the effectiveness of traditional chemotherapy are needed. To our knowledge, this is the first study to demonstrate the potential efficacy of high O_2_ affinity PolyhHbs for tumor oxygenation. Taken together, the results from this study indicate that T-state PolyhHb and R-state PolyhHb are viable candidates to test in future studies aimed at determining whether they can enhance the efficacy of chemotherapy for the treatment of solid tumors. Finally, given that PolyhHb alone reduced tumor growth, it is possible that PolyhHb may have effects beyond tumor oxygenation. Future studies utilizing a form of PolyhHb that is unable to transport O_2_ as a control are warranted. Finally, our studies also showed that the tumor microenvironment (flow rate, capillary density, etc.) had a much greater effect than varying the dose or type of PolyhHb. For example, we postulate that PolyhHbs will be ineffective in a tumor characterized by high blood flow rates and reduced vascular density. Therefore, it would be useful to develop biomarkers in order to probe the tumor microenvironment in order to stratify which patients may benefit from PolyhHb treatment.

## Supporting information

S1 Text Relevant mass transfer with reactions and mesh geometries. Document outlining the mass transfer equations with parameters for our model. An additional description of the mesh geometry is also included.
(PDF)

S1 Dataset Fluid behavior analysis data set. Data set of fluid shear stress as a function of apparent shear rate with corresponding fit parameters.
(XLSX)

S2 Dataset COMSOL Parametric sweep dataset. Parametric sweep data set for each simulated dosing material with full false detection rate statistics.
(XLSX)

S3 Dataset PolyhHb biophysical properties. Measured biophysical parameters of the tense and relaxed state PolyhHbs used in the computational model and animal study.
(XLSX)
