# Supplementary material for: The quaternary state of polymerized human hemoglobin regulates oxygenation of breast cancer solid tumors: A theoretical and experimental study
Source: PLoS One. 2018 Feb 7;13(2):e0191275. doi: 10.1371/journal.pone.0191275 (PMC5802857; doi:10.1371/journal.pone.0191275)
Supplement: S1 Text — Document outlining the mass transfer equations with parameters for our model. An additional description of the mesh geometry is also included. (PDF) [file pone.0191275.s001.pdf]

S1 Text

Relevant Mass Transfer with Reactions and Mesh Geometries

Mathematical Model -- Mass Transport with Reaction Kinetics

The distribution of O_2 and Hb in RBCs will be evaluated with mass transport partial differential equations shown in Eq. 1.

$$v \cdot \nabla C_i = D_i[\nabla^2 C_i] + Rct(C_1 \dots C_n) \quad (1)$$

Where C_i, D_i, and Rct represent the species concentration, diffusivity, and the rate of generation or depletion for O_2, PolyhHb, and Hb in RBCs. The diffusivity of Hb and O_2 in the RBC rich core will be modelled using the expressions developed by Bouwer *et al.* [1]

$$D_{Hb,RBC}[m^2/s] = 9.74\times10^{-7}\left(1-\frac{C_{Hb,RBC}\,[g/dL]}{46}\right)10^{-\frac{C_{Hb,RBC}[g/dL]}{128}} \quad (2)$$

$$D_{O_2,RBC}[m^2/s] = 2.77\times10^{-5}\left(1-\frac{C_{Hb,RBC}\,[g/dL]}{100}\right)10^{-\frac{C_{Hb,RBC}[g/dL]}{119}} \quad (3)$$

The diffusivity of the synthesized PolyhHb will be approximated with the Stokes Einstein relationship at 37 °C as shown in Eq. 4.

$$D_{PolyhHb} = \frac{k_B T}{6\pi\mu r_{PolyhHb}} \quad (4)$$

Where k_B, T, and $r_{PolyhHb}$ are the Boltzmann constant, temperature, and average radius of PolyhHb.

The rate of O_2 consumption in the endothelial vessel wall, interstitial space, and tissue space will be calculated with Michaelis-Menten kinetics as shown in Eq. 5.

$$R_{O_2} = -\frac{V_m pO_2}{k_M + pO_2} \quad (5)$$

Where V_M and k_M are the maximum rate of O_2 consumption and the Michaelis-Menten coefficient respectively. The rate of O_2 offloading for Hb can be expressed by the following system:

$$O_2 + Hb \underset{k_{off}}{\overset{k}{\leftrightarrow}} HbO_2 \quad (6)$$

$$R_{O_2} = k_{off} C_{HbO_2} - k C_{O_2} C_{Hb} \quad (7)$$

At equilibrium no O_2 offloading will occur ($R_{O2} = 0$). In this model we will use the Hill equation to account for the equilibrium saturation of oxygenated Hb as shown in Eq. 8.

$$S_e = \frac{C_{HbO_2,e}}{C_{Hb,total}} = 1 - \frac{C_{Hb,e}}{C_{Hb,total}} = \frac{pO_2^n}{pO_2^n + p50^n} \quad (8)$$

Where pO_2, n, and P_{50} are the partial pressure of O_2, the cooperativity coefficient, and the partial pressure of O_2 at 50% saturation, respectively. Applying the equilibrium assumption to Eq. 7 and rearranging to solve for the rate of O_2 offloading, the following equation can be obtained:

$$R_{Hb-O_2} = k_{off} \mathrm{C}_{\mathrm{Hb}_{\mathrm{total}}} \left(S - \frac{S_e}{1-S_e}(1-S) \right) \tag{9}$$

Where S is the current O_2 saturation of Hb ($C_{HbO2}/C_{Hb,total}$). The physical parameters for the model can be found in Table 1.

Table 1: KTC model parameters.

Symbol	Simulation Parameter	Value	Units	Source
Model Constants				
α	O_2 solubility	1.71×10^{-3}	mol/(m^3·mm Hg)	[2]
ρ_0	Plasma density	1.0	g/cm^3	[3]
κ_M	Endothelial permeability	0.25×10^{-5}	µm^2/(Pa·s)	[4]
κ_T	Tissue and interstitial space permeability	0.64×10^{-2}	µm^2/(Pa·s)	[4]
$D_{O_2,p}$	Diffusivity of O_2 in plasma	1.85×10^{-5}	cm^2/s	[5]
$D_{O_2,e}$	Diffusivity of O_2 in the endothelial cell wall	8.73×10^{-6}	cm^2/s	[5]
$D_{O_2,i}$	Diffusivity of O_2 in the interstitial region	2.81×10^{-5}	cm^2/s	[5]
$D_{O_2,t}$	Diffusivity of O_2 in the tissue space	6.30×10^{-6}	cm^2/s	[5]
K_M	Michaelis constant	5	mm Hg	[2]
L	Length of the arteriole	50	Mm	-
r_E	Thickness of the endothelial cell wall	0.0008	Mm	[6]
r_I	Thickness of the interstitial space	0.0039	Mm	[6]
$H_{t,i}$	Initial hematocrit	40	%	-
Varied Parameters				
v	Average velocity	0.01, 0.1, 0.3, 1	cm/s^2	[6]
V_{max}	Maximum O_2 consumption rate	20, 50, 80	µM/s	[6]
r_C	Radius of the arteriole	10, (5 – 50)	µm	[6]
r_T	Thickness of the tissue space	50, 65, 80	µm	[6]
%TL	Top load percentage	0, 10, 20, 30	%	-
$pO_{2,in}$	Inlet pO_2	1 – 90	mm Hg	-

The biophysical properties of the 35:1 T-state PolyhHb, 30:1 R-state PolyhHb and hHb used in these models were each analyzed in a previous study [7]. Table 2 summarizes the biophysical properties of each species used in this study.

Table 2: Biophysical properties of PolyhHb [7] **and hHbs in RBCs**[8–10]**.**

Property	Accellular hHb	hHb in RBCs	35:1 T-state PolyhHb	30:1 R-state PolyhHb
**Diameter (nm)**	5.5	N/A	93.8	87.1
**P_{50} (mm Hg)**	12.4	29.3	37.4	1.96
**Cooperativity (*n*)**	2.6	2.2	0.79	1.10
**$k_{off, O2}$ (s^{-1})**	37.1	2.0	47.4	24.0
**Concentration (mg/mL)**	> 100	340 (× H_t)	100	100

To simulate the effect of carbogen gas inhalation, we increased the concentration of O_2 available at the inlet by 0.69 mM. Due to Bohr effect, the P_{50} of the hHb in RBCs was increased to 40 mm Hg [11]. When a rhabdomyosarcoma type tumor was modeled, myoglobin was simulated in the tissue region with a D_{Mb} of 2 × 10^{-7} cm^2/s [12], C_{Mb} of 190 μM [13], p50 of 2.8 mm Hg [14], n of 1.10 [15], and k_{off,O_2} of 22 s^{-1} [16]. Myoglobin O_2 kinetics was simulated with equilibrium mediated kinetics from equation 9.

COMSOL Mesh Geometry

The finite element method discretizes the model geometry by creating a mesh with smaller sections (elements) within each region. A custom mesh was implemented in this model to assist with reducing computational times without affecting the results. In short, the mesh was calibrated for predefined "finer" fluid dynamics. A mapped edge with a 3 × 10^{-7} maximum element size was set at the arteriole inlet and calibrated for extremely fine fluid dynamics. Boundary edge layers were defined at the central axis and at the interfaces between each of the layers. Finally, a free triangular structure calibrated for extra fine fluid dynamics was implemented in the tissue space. Images of these mesh structures are found in Fig. 1. The structured mesh pattern in the tissue space shown in (A) and (B) arises from COMSOL's automated mesh construction, which facilitated rapidly meshed geometries for a wide range of arteriole radii and tissue layer thicknesses. Decreased mesh size and smoothing of this region was analyzed within COMSOL and found to have negligible effect on the resulting numerical solutions. The low quality region that exists at the cell free region boundaries results from the application of the boundary edge layers. Inclusion of this layer is necessary to numerically solve the Quemeda characteristic equation without increasing the number of elements in the simulation while maintaining a low computation time.

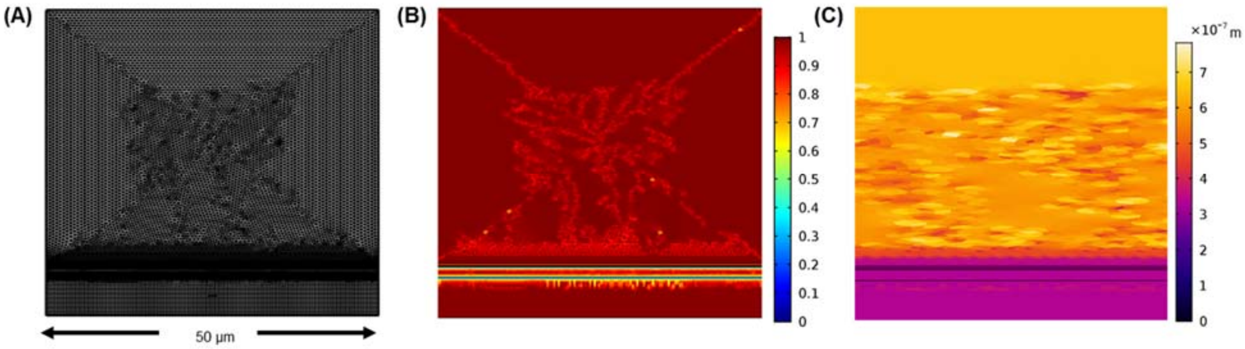

Fig. 1 KTC model COMSOL mesh. (A) Representation of the mesh elements for a single model geometry with a blood vessel radius of 10 µm, tissue layer thickness of 50 µm, and length of 50 µm. (B) The same mesh but with a plot of the element quality where 1 depicts elements with the highest quality and 0 depicts elements with the lowest quality. (C) The same mesh including the size distribution of each element.

Table 3: COMSOL mesh statistics.

COMSOL Mesh Statistics	
Mesh Vertices	23091
Triangular Elements	18119
Quadrilateral Elements	14550
Edge Elements	1181
Vertex Elements	12
Minimum Element Quality	3.596×10^{-4}
Average Element Quality	0.7321
Element Are Ratio	8.717×10^{-5}
Maximum Growth Rate	1.929
Average Growth Rate	1.242

Mesh statisitics for the custom mesh of the tumor arteriole developed in COMSOL Multiphysics with $r_c = 10$ and $r_t = 50$.

Works Cited

1. Bouwer ST, Hoofd L, Kreuzer F. Diffusion coefficients of oxygen and hemoglobin measured by facilitated oxygen diffusion through hemoglobin solutions. Biochim Biophys Acta - Protein Struct Mol Enzymol. 1997;1338: 127–136. doi:10.1016/S0167-4838(96)00197-5

2. Kavanagh BD, Secomb TW, Hsu R, Lin PS, Venitz J, Dewhirst MW. A theoretical model for the effects of reduced hemoglobin-oxygen affinity on tumor oxygenation. Int J Radiat

Oncol Biol Phys. 2002;53: 172–179.

3. Fournier RL. Basic Transport Phenomena in Biomedical Engineering,Third Edition. CRC Press; 2011.

4. Baxter LT, Jain RK. Transport of fluid and macromolecules in tumors. I. Role of interstitial pressure and convection. Microvasc Res. 1989;37: 77–104. doi:10.1016/0026-2862(89)90074-5

5. Vadapalli A, Goldman D, Popel AS. Calculations of Oxygen Transport By Red Blood Cells and Hemoglobin Solutions in Capillaries. Artif Cells, Blood Substitutes, Biotechnol. 2002;30: 157–188. doi:10.1081/BIO-120004338

6. Secomb TW, Hsu R, Dewhirst MW, Klitzman B, Gross JF. Analysis of oxygen transport to tumor tissue by microvascular networks. Int J Radiat Oncol Biol Phys. 1993;25: 481–489. doi:10.1016/0360-3016(93)90070-C

7. Belcher DA, Banerjee U, Baehr CM, Richardson KE, Cabrales P, Berthiaume F, et al. Mixtures of tense and relaxed state polymerized human hemoglobin regulate oxygen affinity and tissue construct oxygenation. Giuffrè A, editor. PLoS One. Humana Press; 2017;12: e0185988. doi:10.1371/journal.pone.0185988

8. Patton JN, Palmer AF. Numerical simulation of oxygen delivery to muscle tissue in the presence of hemoglobin-based oxygen carriers. Biotechnol Prog. 2006;22: 1025–1049. doi:10.1021/bp060022a

9. Sakai H, Tsai AG, Rohlfs RJ, Hara H, Takeoka S, Tsuchida E, et al. Microvascular responses to hemodilution with Hb vesicles as red blood cell substitutes: influence of O2 affinity. Am J Physiol. 1999;276: H553-62. Available: http://www.ncbi.nlm.nih.gov/pubmed/9950857

10. Kavdia M, Popel AS. Wall shear stress differentially affects NO level in arterioles for volume expanders and Hb-based O2 carriers. Microvasc Res. 2003;66: 49–58. Available: http://www.ncbi.nlm.nih.gov/pubmed/12826074

11. Jensen FB. Red blood cell pH, the Bohr effect, and other oxygenation-linked phenomena in blood O2 and CO2 transport [Internet]. Acta Physiologica Scandinavica. Blackwell Science Ltd; 2004. pp. 215–227. doi:10.1111/j.1365-201X.2004.01361.x

12. Papadopoulos S, Endeward V, Revesz-Walker B, Jurgens KD, Gros G. Radial and longitudinal diffusion of myoglobin in single living heart and skeletal muscle cells. Proc Natl Acad Sci U S A. National Academy of Sciences; 2001;98: 5904–9. doi:10.1073/pnas.101109798

13. Swaanenburg JCJM, Visser-Vanbrummen PJ, Dejongste MJL, Tiebosch ATHM. The Content and Distribution of Troponin I, Troponin T, Myoglobin, and alpha-Hydroxybutyric

Acid Dehydrogenase in the Human Heart. Clin Chem Am J Clin Pathol. 2001;115: 770–777. Available: https://pdfs.semanticscholar.org/0b57/e25388d994a6a564f33d7e2aab34d66c03c7.pdf

14. Schenkman KA, Marble DR, Burns DH, Feigl EO. Myoglobin oxygen dissociation by multiwavelength spectroscopy. J Appl Physiol. American Physiological Society; 1997;82: 86–92. Available: http://www.ncbi.nlm.nih.gov/pubmed/9029202

15. Seiyama A. Virtual cooperativity in myoglobin oxygen saturation curve in skeletal muscle in vivo. Dyn Med. BioMed Central; 2006;5: 3. doi:10.1186/1476-5918-5-3

16. Springer ' BA, Sligar SG, Olson ' JS, Phillips GN. Mechanisms of Ligand Recognition in Myoglobin. Chem Rev. 1994;94: 699–714. Available: http://pubs.acs.org/doi/pdf/10.1021/cr00027a007
